# A comparison over 2 decades of disability-free life expectancy at age 65 years for those with long-term conditions in England: Analysis of the 2 longitudinal Cognitive Function and Ageing Studies

**DOI:** 10.1371/journal.pmed.1003936

**Published:** 2022-03-15

**Authors:** Holly Q. Bennett, Andrew Kingston, Ilianna Lourida, Louise Robinson, Lynne Corner, Carol Brayne, Fiona E. Matthews, Carol Jagger, the Cognitive Function and Ageing Studies Collaboration

**Affiliations:** 1 Population Health Sciences Institute, Faculty of Medical Sciences, Newcastle University, Newcastle, United Kingdom; 2 Cambridge Public Health, University of Cambridge, Cambridge, United Kingdom; Harvard Medical School, UNITED STATES

## Abstract

**Background:**

Previous research has examined the improvements in healthy years if different health conditions are eliminated, but often with cross-sectional data, or for a limited number of conditions. We used longitudinal data to estimate disability-free life expectancy (DFLE) trends for older people with a broad number of health conditions, identify the conditions that would result in the greatest improvement in DFLE, and describe the contribution of the underlying transitions.

**Methods and findings:**

The Cognitive Function and Ageing Studies (CFAS I and II) are both large population-based studies of those aged 65 years or over in England with identical sampling strategies (CFAS I response 81.7%, *N =* 7,635; CFAS II response 54.7%, *N* = 7,762). CFAS I baseline interviews were conducted in 1991 to 1993 and CFAS II baseline interviews in 2008 to 2011, both with 2 years of follow-up. Disability was measured using the modified Townsend activities of daily living scale. Long-term conditions (LTCs—arthritis, cognitive impairment, coronary heart disease (CHD), diabetes, hearing difficulties, peripheral vascular disease (PVD), respiratory difficulties, stroke, and vision impairment) were self-reported. Multistate models estimated life expectancy (LE) and DFLE, stratified by sex and study and adjusted for age. DFLE was estimated from the transitions between disability-free and disability states at the baseline and 2-year follow-up interviews, and LE was estimated from mortality transitions up to 4.5 years after baseline. In CFAS I, 60.8% were women and average age was 75.6 years; in CFAS II, 56.1% were women and average age was 76.4 years. Cognitive impairment was the only LTC whose prevalence decreased over time (odds ratio: 0.6, 95% confidence interval (CI): 0.5 to 0.6, *p* < 0.001), and where the percentage of remaining years at age 65 years spent disability-free decreased for men (difference CFAS II–CFAS I: −3.6%, 95% CI: −8.2 to 1.0, *p* = 0.12) and women (difference CFAS II–CFAS I: −3.9%, 95% CI: −7.6 to 0.0, *p* = 0.04) with the LTC. For men and women with any other LTC, DFLE improved or remained similar. For women with CHD, years with disability decreased (−0.8 years, 95% CI: −3.1 to 1.6, *p* = 0.50) and DFLE increased (2.7 years, 95% CI: 0.7 to 4.7, *p* = 0.008), stemming from a reduction in the risk of incident disability (relative risk ratio: 0.6, 95% CI: 0.4 to 0.8, *p* = 0.004). The main limitations of the study were the self-report of health conditions and the response rate. However, inverse probability weights for baseline nonresponse and longitudinal attrition were used to ensure population representativeness.

**Conclusions:**

In this study, we observed improvements to DFLE between 1991 and 2011 despite the presence of most health conditions we considered. Attention needs to be paid to support and care for people with cognitive impairment who had different outcomes to those with physical health conditions.

## Abstract 2

Holly Bennett and colleagues investigate trends in life expectancy and disability-free life expectancy between 1991 and 2011 for adults age 65 years or older with long term health conditions using data from two population-based studies in England.

## Author summary

**Why was this study done?:**

- There have been many advances in healthcare since the 1990s, which means many people with health conditions are living longer lives.
- We wanted to find out whether the extension to life for those with health conditions involves an increase in healthy or unhealthy years.

**What did the researchers do and find?:**

- We used information from 2 large studies of people aged 65 years and over, the first began in 1991 and the second began in 2011.
- We estimated how many years people with different health conditions would live with or without disability in 2011 compared to 1991.
- We found improvements for people with most health conditions, with the exception of cognitive impairment where the increase in years lived with disability were equal (men) or greater than (women) the improvement in years lived without disability.

**What do these findings mean?:**

- In the UK, the percentage of people with cognitive impairment has decreased over time.
- However, if people with cognitive impairment are now living longer with disability than before, this may not mean that the demand for services and care will decrease.

## Introduction

Life expectancy (LE) and disability-free life expectancy (DFLE) have been increasing over time, but this has occurred unequally across the population [1]. While DFLE has improved for the most advantaged men, due to reduced risk of death and increased recovery from disability, and for most advantaged women from reduced incident disability, these trends were not seen for the least advantaged [2]. Moreover the increase in prevalence of multimorbidity, or multiple long-term conditions (MLTCs), and the stronger association between MLTCs and disability for the least advantaged explains this only partially [3]. We are therefore interested in examining which individual long-term conditions (LTCs) have become less disabling, and which might provide the greatest improvement in DFLE if eliminated.

As medical advances and public health practices have contributed to substantial reductions in mortality from leading causes of death such as cardiovascular disease (CVD) [4] and stroke [5], the focus is shifting to the need to improve quality of life and reduce the burden on individuals, health, and social care. This moves the focus from using LE as the measure of success to using healthy or DFLE, the number of years from a particular age spent healthy or free of disability. Comparing the effect of LTCs on DFLE rather than just LE means that fatal and nonfatal conditions can be assessed on the same metric. Not only do we want to enhance health to maximise healthy life years, but if we know which conditions have the greatest impact on DFLE, we can also target resources on delaying the onset of disability and reducing disabling consequences once it does.

An overview of existing literature indicates that most studies on single LTCs and DFLE examine the impact primarily of diabetes, ischaemic heart disease, respiratory diseases, and arthritis, and only at one time point. Temporal comparisons are, as far as we are aware, limited to 3 studies, reporting improvements in remaining healthy years for people with diabetes [6], stroke [7], and CVD [8]. People with diabetes in a US cohort from 2002 had fewer years with disability, more disability-free and total years of life at age 70 years, and became disabled later, compared to those with diabetes in an earlier cohort in 1992 [6]. Similarly, at age 65 years, people with stroke in the later cohort (2000) had longer LE, spent more years disability-free, and fewer years with disability compared to those with stroke in 1992. However, stroke remained an important cause of disability; LE was reduced by 20% to 40% but disability-free years by up to 90% in stroke survivors [7]. The final study considered several LTCs and found improvements from 1990 to 2016 in years gained if CVD was eliminated but similar gains in healthy years in 1990 and 2016 if chronic respiratory diseases, cancer, or diabetes were eliminated [8]. However, there are limitations to these studies. LE and DFLE differ greatly between men and women [9], yet 2 of the studies do not estimate LE and DFLE separately for men and women [6,7]. One study was based on cross-sectional rather than longitudinal data, and, therefore, the underlying transitions between disability states and mortality could not be estimated [8].

Global estimates of trends in disability-adjusted life years (DALYs) suggest that increasing diabetes prevalence has contributed to rising DALY rates and that ischaemic heart disease, stroke, and chronic obstructive pulmonary disease remain the leading causes of DALYs in those aged 50 and older [10]. However, DALYs combine years of life lost (YLL) and years lived with disability (YLD), and thus it is difficult to see whether conditions have had a greater impact on mortality or disability. This is important since elimination of a LTC that has a greater effect on LE than DFLE could increase years spent with disability.

We use longitudinal data from the Cognitive Function and Ageing Studies (CFAS I and II), 2 large population-based studies of people aged 65 years or older in England, to identify trends in LE and DFLE for a wide range of LTCs separately for men and women. Our aims included determining whether people with specific LTCs have experienced longer LE and more years free of disability between 1991 and 2011, and, secondly, which LTCs if eliminated will lead to compression or expansion of disability. In addition, the longitudinal data for each study enables us to explore how the underlying transitions to and from disability, and to death, contribute to the observed trends by LTC.

## Methods

### Data

CFAS I and CFAS II are 2 large population-based studies of people aged 65 years or older living in England [11–13]. Sampling strategy was identical for CFAS I and CFAS II. Individuals were identified through the primary care lists in 3 centres (Newcastle, Nottingham, and Cambridgeshire) and included people living in care settings, semi-dependent housing and in the community. Sampling was stratified for those aged 65 to 74 years, and those aged 75 or above. Baseline interviews were conducted from 1991 to 1993 for CFAS I and from 2008 to 2011 for CFAS II. Follow-up interviews were conducted 2 years later; everyone who participated at baseline and were still alive was reapproached. An informant interview was requested on a subsample of participants. Informant interviews were requested for all those with a Mini Mental State Examination (MMSE) [14] score of 21 or below and a random sample of 10% of the remaining participants, resulting in 16.4% having informants in CFAS I and 11.4% in CFAS II. The participant would nominate a friend or family member who would complete an interview covering the same topics as the participant interview. This information could then be directly substituted for item nonresponse from the participant interview. Date of death was received routinely from the Office for National Statistics (ONS).

### Measures

Demographics included age group (65 to 69 years, 70 to 74 years, 75 to 79 years, 80 to 84 years, 85 to 89 years, and ≥90 years), sex, years in education (<10 years, 10 to 11 years, and ≥12 years), social class based on occupation (skilled, semi-skilled, and unskilled), and place of residence (community, semi-dependent housing, and care settings). Area-level deprivation was measured through the Townsend deprivation index [15], based on information on employment, household overcrowding and car ownership.

Disability was categorised into any disability or disability-free using the modified Townsend activities of daily living (ADL) scale [16,17] and measured at baseline and 2-year follow-up interview. Those who were either housebound (ambulant inside the house, chair bound, or bed bound as opposed to ambulant outside the house) or needed help with one of the following ADL: Washing all over, preparing and cooking a hot meal, putting on shoes and socks, heavy housework or shopping, and carrying heavy bags were considered to be living with disability. If they did not need help with any of the above and were ambulant outside the house, they were classified as disability-free. The original analysis plan (S1 Text) included severity of disability but allowed for mild/moderate and severe disability to be grouped together if numbers were low, which was the case for this analysis.

In total, 9 LTCs were considered, including arthritis, coronary heart disease (CHD—angina or heart attack), cognitive impairment, diabetes, hearing difficulties, peripheral vascular disease (PVD), respiratory difficulties (asthma except childhood only or chronic bronchitis), stroke, and vision impairment, based on previous analysis [18]. All were self-reported apart from cognitive impairment, which was defined as a score less than 26 on the MMSE. Hearing difficulties and vision impairment were both self-reported as well as the interviewer rating whether they had problems with their sight or hearing. Item nonresponse was low and ranged from 0.3% for hearing difficulties to 2.1% for CHD in CFAS I and between 0.8% for hearing difficulties and 3.8% for CHD in CFAS II. We defined MLTCs as the presence of 2 or more health conditions. For those individuals with missing health conditions, MLTCs was determined if the percentage of measured health conditions exceeded 22.2% (equivalent to 2 out of 9). Questions used to determine presence of health conditions and disability are included in S2 Text.

### Statistical analysis

Demographics were inverse probability weighted for nonresponse. The nonresponse weights included age, sex, deprivation, and whether the participant lived in care settings. Health condition prevalence from CFAS I and CFAS II was weighted for nonresponse and age and sex standardised to the CFAS I population (1991). We used logistic regression to compare the prevalence of each LTC at baseline between the 2 studies and the extent to which age group, sex, and time contributed to differences in prevalence. All logistic regression models were weighted for nonresponse.

LEs were estimated from longitudinal multistate models analysing transitions from disability-free to disability, recovery from disability to disability-free, and from either disability state to death in Interpolated Markov Chain (IMaCh) software version 0.99r19 [19]. IMaCh models discrete time steps, using multinomial logistic regression to model transition probabilities within each step (see S3 Text for further details). Initially length of time between interviews was used as the discrete time step (2 years, 24 months); however, where possible, this was decreased to 1-month steps to approximate continuous time. LE models were stratified by sex and study, with health condition as a covariate. To estimate risk of transitioning between states in CFAS II compared to CFAS I, models were stratified by sex and having a LTC, with study as the covariate. All models converged at 1-month steps with the exception of the women’s stroke and PVD models for between study comparisons that converged at 12-month steps. The models were inverse probability weighted for participants included in the model. Those alive at the censoring date, but who participated only at baseline, were excluded from the multistate models, as they made no recorded transitions. However, those excluded were more likely to have severe disability, which could lead to overestimation of recovery and underestimation of mortality from disability. To account for this, additional weighting was applied to those who were still included in the model by comparing on key variables those who were alive by the censoring date and participated at baseline and 2-year follow up to those excluded (for further details, see S3 Text). As participants in CFAS I and CFAS II were healthy cohorts, an additional weight was also applied to those who died, comparing probability of death in CFAS to probability of death of similar generations from the ONS (see S3 Text for further details). LE was modelled on date of death, and for comparability between CFAS I and II, date of death was included up to 4.5 years after baseline. DFLE and life expectancy with disability (DLE) were estimated from transitions between disability-free and disability states between baseline and 2-year follow-up interviews.

This study is reported as per the Strengthening the Reporting of Observational Studies in Epidemiology (STROBE) guideline (S4 Text).

### Ethics approval

This study was conducted as secondary data analysis of the Cognitive Function and Ageing Studies. The current ethics for MRC CFAS (including CFAS I centres) is from Eastern MREC, reference number 05/MRE05/37 and for the mortality data Wales REC 7, reference number 14/WA/1154. The current REC reference number for CFAS II is 07/MRE05/48 from Cambridge REC 4. For further information on past ethical approvals, please visit the CFAS website (www.cfas.ac.uk).

## Results

### Demographics

In CFAS I, there were 7,635 participants at baseline, 60.8% were women, and average age was 75.6 years. Before the 2-year follow-up interview, 10.7% (*n =* 819) had died, and of those still alive, 76% (5,156/6,816) participated in the 2-year follow-up interview, the remaining 1,660 having moved away or refused. Prevalence of disability at baseline in CFAS I was 31.5% (missing 1.1%), and 37.4% (missing 2.8%) of those who participated at 2-year follow-up had disability. Total person-years for the model was 28,930.4 years, on average 4.5 years in CFAS I. Of the 7,762 participants at baseline in CFAS II, 56.1% were women and average age was 76.4 years. A lower percentage of baseline participants in CFAS II died before the 2-year follow-up interview (8.3%, *n =* 643), and out of the 7,119 people who were still alive, 74% (*n* = 5,288) agreed to another interview, with 1,831 refusing or having moved away. At baseline in CFAS II, 36.4% (missing 4.0%) had disability, and of those who participated at 2-year follow-up, 36.6% (missing 0.7%) had disability. For CFAS II, total person-years was 30,027.8 years, 4.7 years on average. Table 1 gives information on further demographics.

**Table 1:** Number and weighted percentage of demographics at baseline in the CFAS I and CFAS II.

		CFAS I		CFAS II	
		n	%	n	%
Age group (years)	65–69	1,981	25.0	1,939	23.0
	70–74	1,776	22.8	1,873	22.7
	75–79	1,725	22.5	1,624	20.5
	80–84	1,308	17.7	1,278	17.5
	85–89	615	8.5	737	10.5
	≥90	230	3.5	311	5.8
Sex	Men	3,045	39.2	3,534	43.9
	Women	4,590	60.8	4,228	56.1
Education (years)	<10	5,529	74.1	2,047	29.4
	10–11	1,238	16.6	3,923	50.2
	≥12	692	9.3	1,667	20.4
Social class	Skilled	1,921	26.2	1,958	25.4
	Semi-skilled	3,855	52.6	3,962	54.3
	Unskilled	1,555	21.3	1,370	20.2
Place of residence	Community	6,599	86.0	7,083	89.5
	Semi-dependent	683	9.1	482	7.2
	Care settings	346	4.8	197	3.3
Deprivation tertiles	Least	2,561	33.5	2,940	33.2
	Mid	2,525	33.2	2,659	33.3
	Most	2,549	33.4	2,163	33.5
Health condition count	0–1	3,523	45.7	3,420	41.9
	2+	4,102	54.3	4,311	58.1

CFAS, Cognitive Function and Ageing Study.

### Change in prevalence of LTCs between 1991 and 2011

The prevalence of most LTCs in those aged 65 years and over increased between 1991 and 2011, with the prevalence of diabetes and PVD more than doubling (Table 2, Table A in S5 Text). Cognitive impairment was the only condition whose prevalence decreased (odds ratio (OR): 0.6, 95% CI: 0.5 to 0.6, *p* < 0.001) (Table 2). For CHD (OR: 1.3, 95% CI: 1.2 to 1.4, *p* < 0.001), diabetes (OR: 2.5, 95% CI: 2.3 to 2.8, *p* < 0.001), hearing difficulties (OR: 1.2, 95% CI: 1.1 to 1.3, *p* < 0.001), PVD (OR: 2.3, 95% CI: 2.0 to 2.6, *p* < 0.001), and cognitive impairment, these changes were not solely a result of differences in the age and sex distributions of the 2 study populations (Table 2). Moreover, the increases in prevalence of CHD, diabetes, and PVD, and the decrease in prevalence of cognitive impairment, were observed across all age groups (Table A in S5 Text). In order to understand how the presence of other conditions might affect changes in the prevalence of individual LTCs between 1991 and 2011, within those who had at least one LTC, we investigated the percentage who had at least one other LTC. For those with an individual LTC, the percentage of those with at least one other LTC was similarly high in both 1991 and 2011 (Table B in S5 Text).

**Table 2:** OR comparing prevalence of health conditions in the CFAS II to CFAS I, with 95% CIs and *p*-values (p). Models adjusted for sex and age group.

	Unadjusted			Adjusted for age and sex		
	OR	95% CI	*p*	OR	95% CI	*p*
Arthritis	1.1	(1.0, 1.2)	0.002	1.1	(1.1, 1.2)	<0.001
Cognitive impairment	0.7	(0.6, 0.7)	<0.001	0.6	(0.5, 0.6)	<0.001
CHD	1.4	(1.2, 1.5)	<0.001	1.3	(1.2, 1.4)	<0.001
Diabetes	2.6	(2.3, 2.9)	<0.001	2.5	(2.3, 2.8)	<0.001
Hearing difficulties	1.3	(1.2, 1.4)	<0.001	1.2	(1.1, 1.3)	<0.001
PVD	2.3	(2.1, 2.6)	<0.001	2.3	(2.0, 2.6)	<0.001
Respiratory difficulties	1.1	(1.0, 1.2)	0.04	1.1	(1.0, 1.2)	0.03
Stroke	1.3	(1.2, 1.4)	<0.001	1.2	(1.1, 1.4)	<0.001
Vision impairment	1.2	(1.1, 1.3)	<0.001	1.2	(1.1, 1.3)	0.002

CFAS, Cognitive Function and Ageing Study; CHD, coronary heart disease; CI, confidence interval; OR, odds ratio; PVD, peripheral vascular disease.

### Life expectancy and disability-free life expectancy at age 65 years with each LTC

**Fig 1:**
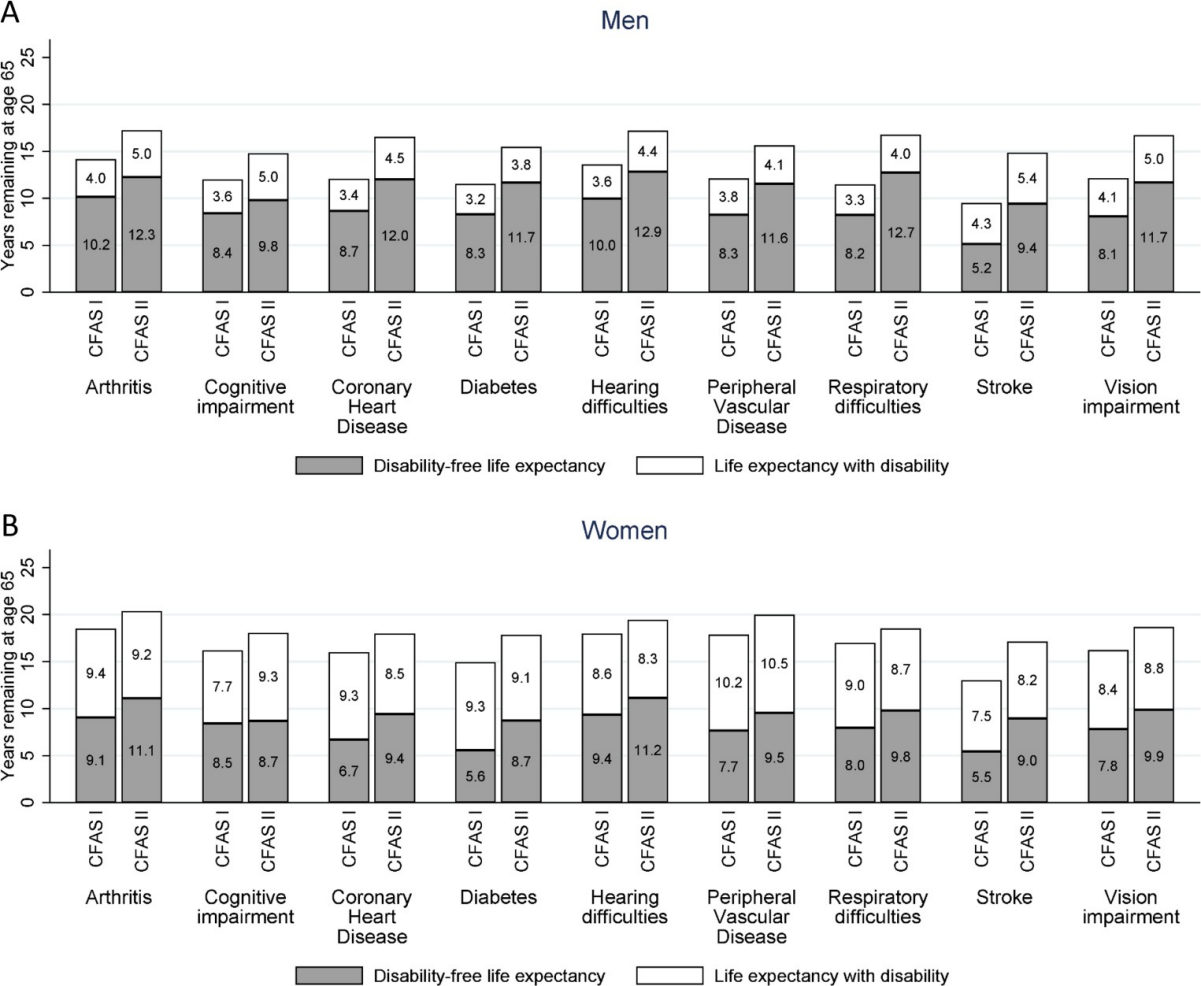
DFLE and DLE at age 65 years for men (A) and women (B) with a health condition in the CFAS I and CFAS II. Models stratified by sex and study and adjusted for age and the health condition. Results also shown in Tables C and E in S5 Text with 95% CIs and *p*-values. CFAS, Cognitive Function and Ageing Study; DFLE, disability-free life expectancy; DLE, life expectancy with disability.

We have previously reported that in the period between CFAS I and CFAS II, men’s LE at age 65 years increased by 4.6 years (95% confidence interval (CI): 3.7 to 5.5 years, *p*-value (p) < 0.001) of which the majority (3.7 years, 95% CI: 2.7 to 4.8 years, *p* < 0.001) were years free of disability [2]. For men without any of the health conditions considered their LE at age 65 years in CFAS I was 18.1 years (95% CI: 16.2 to 20.0) with a DFLE of 14.1 years (95% CI: 12.2 to 15.9). In CFAS II, men without any of the health conditions had a LE of 20.6 years (95% CI: 18.8 to 22.3) and DFLE of 18.0 years (95% CI: 16.4 to 19.6). Thus, men aged 65 without any health conditions gained 2.5 years in LE (95% CI: −0.1 to 5.0, *p* = 0.06) and 3.9 years in DFLE (95% CI: 1.5 to 6.3, *p* = 0.001). In the presence of LTCs, there was a greater gain in DFLE than DLE (Fig 1A, Table C in S5 Text) but the proportion of time lived disability-free remained similar (Table D in S5 Text). The exception was for men with stroke where gains in DFLE far outweighed gains in DLE, resulting in an improvement in the percentage of life spent disability-free from 54.3% in 1991 to 63.5% in 2011 (Table D in S5 Text, difference 9.2%, 95% CI: 1.4 to 17.0, *p* = 0.02). The greatest improvements in DFLE were seen for men with respiratory difficulties (4.5 years, 95% CI: 2.6 to 6.4, *p* < 0.001) and men living poststroke (4.3 years, 95% CI: 1.8 to 6.8, *p* < 0.001) (Fig 1A). Men with cognitive impairment experienced the smallest increase in DFLE (1.4 years, 95% CI: −0.7 to 3.4, *p* = 0.18), and a similar level of increase in DLE (1.4 years, 95% CI: 0.2 to 2.5, *p* = 0.02), despite cognitive impairment being the only health condition to reduce in prevalence.

Between CFAS I and II, women experienced an increase in LE at age 65 years of 2.1 years (95% CI: 1.1 to 3.0 years, *p* < 0.001), with an almost equal increase in DFLE of 2.0 years (95% CI: 1.0 to 2.9 years, *p* < 0.001) [2]. Women without any of the health conditions at age 65 years had a LE of 21.8 years (95% CI: 19.3 to 24.3) and could expect 15.3 of those years (95% CI: 13.1 to 17.4) to be disability-free in CFAS I. By CFAS II, LE reached 23.9 years (95% CI: 21.1 to 26.8) with 17.0 years DFLE (95% CI: 15.5 to 18.6). Women aged 65 without any health conditions gained 2.1 years LE (95% CI: −1.6 to 5.9, *p* = 0.26) and 1.8 years DFLE (95% CI: −0.9 to 4.4, *p* = 0.20). Similar to men, most improvement in LE at age 65 years for women with each LTC was in disability-free years. While there was no reduction in DLE for men with health conditions, women with some conditions did see a reduction in DLE (Fig 1B, Table E in S5 Text). For example, women with CHD experienced a decline in DLE (−0.8 years, 95% CI: −3.1 to 1.6, *p* = 0.50) (Fig 1B), resulting in an increase in percentage of remaining years spent disability-free (CFAS I: 42.1%, CFAS II: 52.6%, difference 10.5%, 95% CI: 5.2 to 15.8, *p* < 0.001) (Table F in S5 Text). The largest gains in DFLE occurred in women with stroke (3.5 years, 95% CI: 0.4 to 6.6, *p* = 0.03) (Fig 1B, Table E in S5 Text), but this gain was not as large as for men (4.3 years). Women with cognitive impairment experienced a large increase in DLE (1.6 years, 95% CI: 0.1 to 3.1, *p* = 0.04) without any improvement in DFLE (Fig 1B, Table E in S5 Text). Consequently, the percentage of remaining years disability-free decreased for women with cognitive impairment (CFAS I: 52.2%, CFAS II: 48.3%, difference −3.9%, 95% CI: −7.6 to 0.0, *p* = 0.04) (Table F in S5 Text).

### Probability of transitioning between disability states and death by LTC

**Fig 2:**
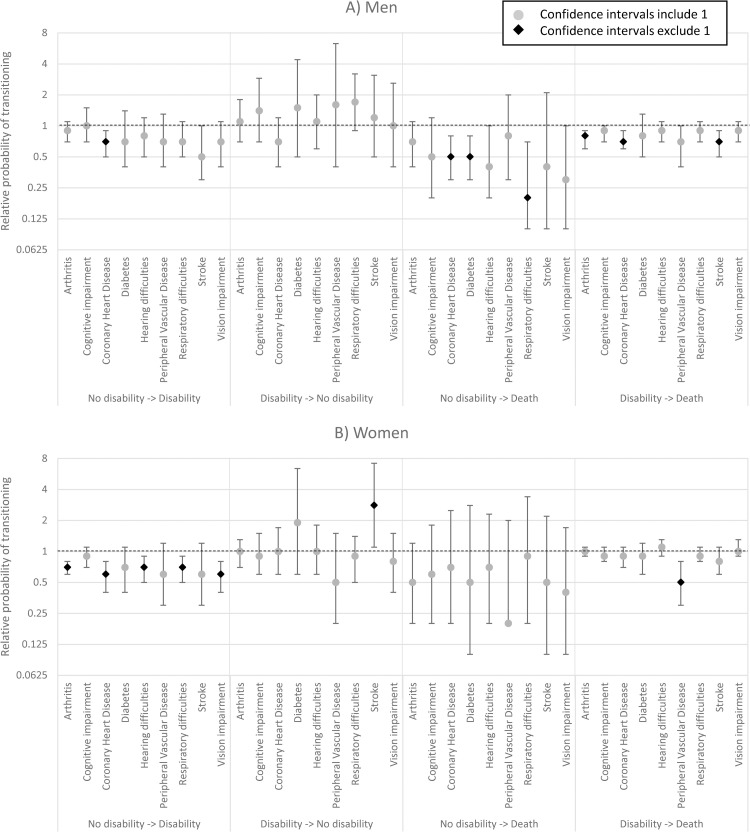
RRR of transitioning between disability states in the CFAS II (2011) compared to CFAS I (1991) for men and women with each LTC with 95% CI. Estimates from models stratified by sex and health condition with age and study covariates. Results also shown in Table 3 with 95% CI and *p*-values. CFAS, Cognitive Function and Ageing Studies; CI, confidence interval; RRR, relative risk ratio; LTC, long-term condition.

The large improvements in DFLE seen in men with respiratory difficulties (4.5 years) appear to be a result of a decrease in the probability of death from a disability-free state (relative risk ratio (RRR): 0.2, 95% CI: 0.1 to 0.7, *p* = 0.001; Fig 2A, Table 3). Similar sized improvements in DFLE in men with stroke (4.3 years) potentially resulted from a decrease in the probability of death from a disability state (RRR: 0.7, 95% CI: 0.5 to 0.9, *p* = 0.02), although the probability of incident disability was halved for men with stroke between CFAS I and CFAS II (RRR: 0.5, 95% CI: 0.3 to 1.0, *p* = 0.02; Fig 2A, Table 3). Men with cognitive impairment experienced the smallest increase in DFLE (1.4 years) with the same sized increase in DLE (men with other health conditions having increase in DFLE greater than increase in DLE), potentially from apparent but not statistically significant reductions in the probability of death from either disability-free (RRR: 0.5, 95%CI: 0.2 to 1.2, *p* = 0.13) or with disability (RRR: 0.9, 95% CI: 0.7 to 1.0, *p* = 0.25), without improvement in any other transitions.

**Table 3:** RRR of transitioning between disability states in the CFAS II (2011) compared to CFAS I (1991) for men and women with each LTC, with 95% CIs and *p*-values (p). Estimates from models stratified by sex and health condition with age and study covariates.

		Men			Women^1^		
		RRR	95% CI	*p*	RRR	95% CI	*p*
No disability -> Disability	Arthritis	0.9	(0.7, 1.1)	0.36	0.7	(0.6, 0.8)	<0.001
	Cognitive impairment	1.0	(0.7, 1.5)	0.91	0.9	(0.7, 1.1)	0.36
	CHD	0.7	(0.5, 0.9)	0.02	0.6	(0.4, 0.8)	0.004
	Diabetes	0.7	(0.4, 1.4)	0.26	0.7	(0.4, 1.1)	0.17
	Hearing difficulties	0.8	(0.5, 1.2)	0.32	0.7	(0.5, 0.9)	0.02
	PVD	0.7	(0.4, 1.3)	0.24	0.6	(0.3, 1.2)	0.15
	Respiratory difficulties	0.7	(0.5, 1.1)	0.08	0.7	(0.5, 0.9)	0.02
	Stroke	0.5	(0.3, 1.0)	0.02	0.6	(0.3, 1.2)	0.15
	Vision impairment	0.7	(0.4, 1.1)	0.17	0.6	(0.4, 0.8)	0.004
Disability -> No Disability	Arthritis	1.1	(0.7, 1.8)	0.69	1.0	(0.7, 1.3)	0.82
	Cognitive impairment	1.4	(0.7, 2.9)	0.35	0.9	(0.6, 1.5)	0.65
	CHD	0.7	(0.4, 1.2)	0.20	1.0	(0.6, 1.7)	0.99
	Diabetes	1.5	(0.5, 4.4)	0.46	1.9	(0.6, 6.4)	0.29
	Hearing difficulties	1.1	(0.6, 2.0)	0.76	1.0	(0.6, 1.8)	0.94
	PVD	1.6	(0.4, 6.3)	0.50	0.5	(0.2, 1.5)	0.18
	Respiratory difficulties	1.7	(0.9, 3.2)	0.10	0.9	(0.5, 1.4)	0.69
	Stroke	1.2	(0.5, 3.1)	0.70	2.8	(1.1, 7.2)	0.03
	Vision impairment	1.0	(0.4, 2.6)	0.93	0.8	(0.4, 1.5)	0.51
No disability -> Death	Arthritis	0.7	(0.4, 1.1)	0.17	0.5	(0.2, 1.2)	0.13
	Cognitive impairment	0.5	(0.2, 1.2)	0.13	0.6	(0.2, 1.8)	0.36
	CHD	0.5	(0.3, 0.8)	0.006	0.7	(0.2, 2.5)	0.58
	Diabetes	0.5	(0.3, 0.8)	0.006	0.5	(0.1, 2.8)	0.41
	Hearing difficulties	0.4	(0.2, 1.0)	0.03	0.7	(0.2, 2.3)	0.57
	PVD	0.8	(0.3, 2.0)	0.64	0.2	(0.0, 2.0)	0.17
	Respiratory difficulties	0.2	(0.1, 0.7)	0.001	0.9	(0.2, 3.4)	0.88
	Stroke	0.4	(0.1, 2.1)	0.24	0.5	(0.1, 2.2)	0.38
	Vision impairment	0.3	(0.1, 1.0)	0.04	0.4	(0.1, 1.7)	0.20
Disability -> Death	Arthritis	0.8	(0.6, 0.9)	0.03	1.0	(0.9, 1.1)	0.86
	Cognitive impairment	0.9	(0.7, 1.0)	0.25	0.9	(0.8, 1.1)	0.19
	CHD	0.7	(0.6, 0.9)	<0.001	0.9	(0.7, 1.1)	0.36
	Diabetes	0.8	(0.5, 1.3)	0.36	0.9	(0.6, 1.2)	0.55
	Hearing difficulties	0.9	(0.7, 1.1)	0.36	1.1	(0.9, 1.3)	0.31
	PVD	0.7	(0.4, 1.0)	0.13	0.5	(0.3, 0.8)	0.006
	Respiratory difficulties	0.9	(0.7, 1.1)	0.36	0.9	(0.8, 1.1)	0.19
	Stroke	0.7	(0.5, 0.9)	0.02	0.8	(0.6, 1.1)	0.15
	Vision impairment	0.9	(0.7, 1.1)	0.36	1.0	(0.9, 1.3)	0.64

^1^Models converged at 1-month steps apart from the women’s PVD and stroke models, which converged at 12-month steps.

CFAS, Cognitive Function and Ageing Studies; CHD, coronary heart disease; CI, confidence interval; LTC, long-term condition; PVD, peripheral vascular disease; RRR, relative risk ratio.

Men with arthritis (RRR: 0.8, 95% CI: 0.6 to 0.9, *p* = 0.03) and CHD (RRR: 0.7, 95% CI: 0.6 to 0.9, *p* < 0.001) also experienced reductions in the probability of death from the disability state. In the case of CHD (RRR: 0.7, 95% CI: 0.5 to 0.9, *p* = 0.02) and stroke (RRR: 0.5, 95% CI: 0.3 to 1.0, *p* = 0.02), but not in men with cognitive impairment, there was also a reduction in the probability of incident disability (Fig 2A, Table 3). Along with men with respiratory difficulties, men with CHD (RRR: 0.5, 95% CI: 0.3 to 0.8, *p* = 0.006) or diabetes (RRR: 0.5, 95% CI: 0.3 to 0.8, *p* = 0.006) also experienced a reduction in the probability of death from a disability-free state.

Women with CHD experienced a decline in DLE (−0.8 years), possibly because of a decline in the likelihood of transitioning to disability (RRR: 0.6, 95% CI: 0.4 to 0.8, *p* = 0.004) (Fig 2B, Table 3). In addition, women with arthritis (RRR: 0.7, 95% CI: 0.6 to 0.8, *p* < 0.001), hearing difficulties (RRR: 0.7, 95% CI: 0.5 to 0.9, *p* = 0.02), respiratory difficulties (RRR: 0.7, 95% CI: 0.5 to 0.9, *p* = 0.02), or vision impairment (RRR: 0.6, 95% CI: 0.4 to 0.8, *p* = 0.004) were less likely to transition to disability in CFAS II compared to CFAS I (Fig 2B, Table 3).

The largest increase in DFLE occurred in women with stroke (3.5 years), and this may be partly explained by the observed substantial increase in the probability of recovery (transition from disability to no disability), though CIs are wide (RRR: 2.8, 95% CI: 1.1 to 7.2, *p* = 0.03; Fig 2B, Table 3). Women with cognitive impairment experienced no improvement in DFLE between CFAS I and CFAS II and an increase of 1.6 years with disability, although there was no evidence of significant increases or reductions in any of the transitions (Fig 2B, Table 3). For women with LTCs, the only evidence of differences in the probability of death across the studies was for women with PVD where the probability of death with disability halved between the studies (RRR: 0.5, 95% CI: 0.3 to 0.8, *p* = 0.006) (Fig 2B, Table 3).

### Population impact on compression or expansion of morbidity from elimination of individual LTCs

Comparing LE and DFLE for men and women with and without each LTC provides understanding of the impact on population health of eliminating each condition. More specifically, if the gains in DFLE from elimination of a condition (calculated by subtracting DFLE of those with the condition from the DFLE of those without the condition) exceeds the gains in LE, then elimination will lead to a compression of disability; if gains in LE exceed those in DFLE, then expansion of disability will result. We focus on the most recent period (CFAS II) to investigate the population impact of elimination of each LTC, and examine whether elimination of the LTC would result in the years with disability (DLE) significantly decreasing (compression) or increasing (expansion) if the LTC was eliminated.

**Fig 3:**
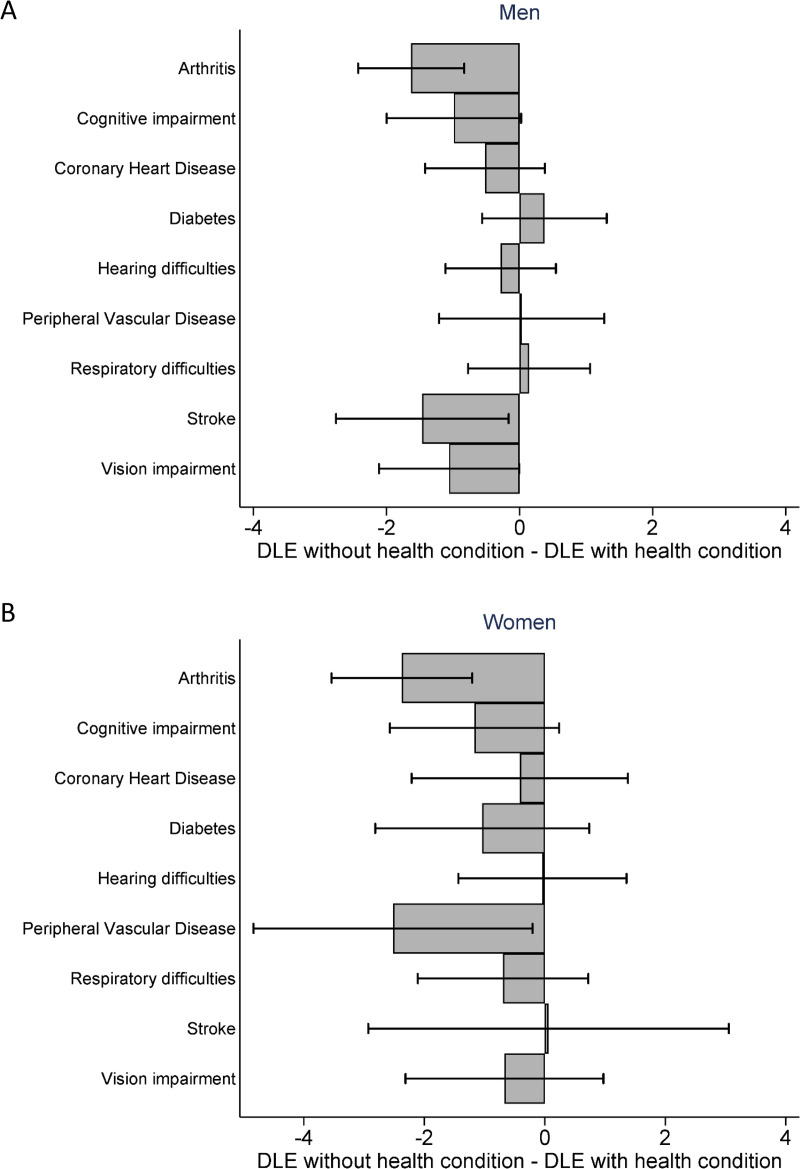
Difference in years with disability (DLE) between men (A) and women (B) with and without the health condition (DLE without health condition–DLE with health condition) in CFAS II with 95% CIs. Models stratified by sex and study and adjusted for age and health condition. Results also shown in Tables C and E in S5 Text with 95% CIs and *p*-values. CFAS, Cognitive Function and Ageing Studies; CI, confidence interval; DLE, life expectancy with disability.

From CFAS II, elimination of arthritis (−1.6 years, 95% CI: −2.4 to −0.8, *p* < 0.001), or stroke (−1.5 years, 95% CI: −2.8 to −0.2, *p* = 0.02) in men could reduce years with disability and therefore result in a compression of disability (Fig 3A, Table C in S5 Text). Compared to other health conditions, LE (3.8 years, 95% CI: 2.3 to 5.3, *p* < 0.001) and DFLE (4.8 years, 95% CI: 3.0 to 6.6, *p* < 0.001) gains would be greatest if cognitive impairment was eliminated and would be expected to result in a compression of disability (−1.0 years, 95% CI: −2.0 to 0.0, *p* = 0.05) (Fig 3A, Table C in S5 Text). For women in CFAS II, elimination of arthritis (−2.4 years, 95% CI: −3.5 to −1.2, *p* < 0.001) or PVD (−2.5 years, 95% CI: −4.8 to −0.2, *p* = 0.03) would be expected to compress disability (Fig 3B, Table E in S5 Text). Although not significant, elimination of cognitive impairment could reduce years with disability (−1.2 years, 95% CI: −2.6 to 0.2, *p* = 0.09; Fig 3B, Table E in S5 Text). By CFAS II, the difference in DLE between women with and without CHD, hearing difficulties or stroke was minimal (Table E in S5 Text). In CFAS I, only elimination of arthritis for both men (−0.8 years, 95% CI: −1.5 to −0.1, *p* = 0.03) and women (−3.2 years, 95% CI: −4.2 to −2.2, *p* < 0.001) could result in a reduction in years with disability and a compression of disability.

To better understand why elimination of an LTC would contribute to compression of disability, we examined the RRRs for those with each condition (compared to those without the condition), separately for men and women and by study.

In CFAS II, cognitive impairment was the largest contributor to loss of years in men’s LE and DFLE (Table C in S5 Text). In comparison to men without cognitive impairment, those with cognitive impairment were more likely to become disabled (RRR: 1.8, 95% CI: 1.3 to 2.5, *p* < 0.001) and less likely to recover (RRR: 0.5, 95% CI: 0.3 to 0.7, *p* = 0.001) from disability (Table G in S5 Text). This also applied to men with stroke (incident disability RRR: 2.0, 95% CI: 1.3 to 3.0, *p* = 0.001; recovery RRR: 0.6, 95% CI: 0.3 to 0.9, *p* = 0.07) compared to men without stroke (Table G in S5 Text). For men with arthritis, however, loss of DFLE in comparison to those without arthritis resulted from an increased probability of transitioning to disability (RRR: 1.6, 95% CI: 1.2 to 2.0, *p* < 0.001) and a decreased probability of death with disability (RRR: 0.7, 95% CI: 0.6 to 0.8, *p* < 0.001; Table G in S5 Text).

For women in CFAS II, DLE was increased for those with arthritis or PVD in comparison to those without the LTC. Women with arthritis were more likely to become disabled (RRR: 1.6, 95% CI: 1.3 to 2.0, *p* < 0.001) than women without arthritis and women with PVD were less likely to die with disability (RRR: 0.7, 95% CI: 0.6 to 0.9, *p* < 0.001) than those without PVD (Table H in S5 Text). The largest contributors to loss of years disability-free for women were cognitive impairment followed by diabetes and stroke. Women with cognitive impairment were not only more likely to become disabled (RRR: 1.5, 95% CI: 1.2 to 1.9, *p* < 0.001) and less likely to recover from disability (RRR: 0.6, 95% CI: 0.4 to 0.8, *p* = 0.004) compared to women without cognitive impairment (the same as men with cognitive impairment) but additionally more likely to die with disability (RRR: 1.2, 95% CI: 1.1 to 1.4, *p* = 0.003; Table H in S5 Text). Women with diabetes (RRR: 1.6, 95% CI: 1.2 to 2.2, *p* = 0.002) or stroke (RRR: 1.7, 95% CI: 1.0 to 2.7, *p* = 0.04) were more likely to become disabled (Table H in S5 Text).

## Discussion

Although LTCs have been reported as major drivers of disability, to the best of our knowledge, our study is the first to quantify the contribution of chronic conditions to trends in DFLE using longitudinal data. We used 2 large, population-representative studies to examine temporal trends in LE and DFLE for those with LTCs to discover whether LTCs are becoming more or less disabling or fatal. Cognitive impairment was the only LTC where the prevalence decreased between 1991 and 2011, but also the only LTC where the percentage of remaining years with disability increased for men and women. Other LTCs showed improvements for both men and women with the health condition, with the majority of LE gains being years free of disability. This was especially true for women with CHD where improvements to LE and DFLE occurred alongside decreases in DLE, providing evidence that compression of disability while increasing life span could be possible in the presence of health conditions. Positive trends in DFLE for men resulted from reductions in the probability of death with or without disability, whereas for women, the trends mainly resulted from reductions in the probability of incident disability.

Although it may not be achievable to fully eliminate a health condition, by comparing DLE of men and women with and without each LTC in 2011, we were able to theoretically explore whether elimination of the LTC would result in a reduction of years with disability (compression of disability). We found that arthritis in men and women, stroke and cognitive impairment in men, and PVD in women, if eliminated, could all result in a significant reduction in years with disability and therefore a compression in disability.

### Context

The increasing prevalence of LTCs, particularly stroke and diabetes, in the last decades has already been documented, at least in the UK and the US [20,21], and is not simply a result of the ageing of populations [22]. Additionally, both the UK and US, and others, report decreasing prevalence and incidence of cognitive impairment and dementia, now a consistent finding across high-income countries where it has been possible to examine trends [23–26]. Whether disability-free LE trends are improving (with compression of disability) or not (expansion of disability) is more controversial. Previous work found increases in DFLE at age 65 years for both men and women, and increases in years with disability for men. These were due to decreases in the probability of developing disability for men and women, and a 50% lower risk of death from no disability for men [2]. However, these increases occurred unequally across the population, and, although MLTCs contributed, they did not fully account for the inequalities [3].

Of the LTCs we considered, the only one for which prevalence has decreased is cognitive impairment. Despite this, the negative association between cognitive impairment and DFLE appeared greater in CFAS II than in CFAS I. This could be due to the greater prevalence of other LTCs being present in those with cognitive impairment in CFAS II compared to CFAS I, although this amounted to only 5 or 6 percentage points on an already large proportion (over 80%) of those with cognitive impairment having MLTCs. Similar results have been reported for changes in comorbidity with dementia in CFAS I and CFAS II [27]. Other studies on cognitive impairment and DFLE have not considered temporal trends but do report a reduction in LE and DFLE for those with cognitive impairment in comparison to those without [18,28,29]. Given the association between higher education and slower cognitive decline [30] and the widening inequalities between education groups in life and health expectancy [1], there may be an interaction between education, cognitive impairment, and the temporal trends in DFLE. However, there may be other reasons for the difference in findings for cognitive impairment compared to the other physical health conditions. For example, there may be differences in the mechanism that causes someone with cognitive impairment to start losing ADLs [31] or from differences in health and social care for people with mental health conditions in the UK. Recent research in England and Wales found that, despite a decade long national policy focus on dementia [32,33], considerable geographical inequalities in postdiagnostic dementia care persist with support services largely focused in the early stages of dementia, tapering off as the illness progresses and thus missing key opportunities to minimise disability via reablement and rehabilitation interventions [34,35].

We found in both CFAS I and II that elimination of arthritis could increase DFLE. This is consistent with other studies reporting lower DFLE, higher DLE [36,37], or greater percentage of remaining years spent with disability [28,37] for men and women with arthritis in comparison to those without arthritis. Again, to our knowledge, there has been no previous work on temporal trends.

While the majority of the literature on the impact of health conditions on life and health expectancy focuses on DALYs, and since DALYs combine years with disability and life years, this does not allow for estimation of the different probabilities of incident disability or recovery from disability at different ages, one of the advantages of estimating DFLE from longitudinal data. For comparability, therefore, we focus on literature reporting trends in DFLE. Similar to our findings, other studies have found improvements in stroke [7] and diabetes [6]. Another study considered healthy years for the whole population (including everyone who did or did not have individual LTCs) and then compared it to healthy years if an individual LTC was eliminated [8]. For men and women, healthy years increased if CVDs, chronic respiratory diseases, cancer, and, to a lesser extent, diabetes were eliminated. They also reported that the increase in healthy years from elimination of CVD was larger in 2016 than in 1990 for men and women. This differs to our findings where the gap in DFLE between those with and without CHD became smaller over time rather than larger, although this might be because of differences in the health expectancy measure, as well as the LTC.

### Strengths and limitations

CFAS I and II have identical sampling frames so are well placed to provide temporal comparisons, giving accurate estimates of changes over 2 decades without compromising the validity of results. Both CFAS I and CFAS II are large population-based studies, which meant that estimates of LE and DFLE could be stratified by sex and a broad range of health conditions could be considered even when prevalence was relatively low. Both studies included residents of care homes and assisted living facilities, important given the difference in prevalence of cognitive impairment in these places of residence. Item nonresponse from the participant interview could be substituted with information from an informant interview with a friend or family member. There were some limitations to this analysis. The presence of health conditions depends on self-report by the participant, which therefore relies on their memory and accuracy of reporting but also on definition and diagnostic practice for the condition. With regard to participant memory, missing information from the participant was substituted by information given by informants to limit the loss of data from this in both CFAS I and CFAS II, resulting in less than 4% missing data in every health condition for both studies. Changes in definition and diagnostic practice have occurred for both stroke and diabetes [38], and, through the Quality Outcomes Framework, incentives for GP practices to diagnose and appropriately treat certain conditions including hypertension, CVD, stroke, and diabetes [39]. Although 24 is usually used as a cut point for cognitive impairment in the MMSE, the DFLE models with lower cut points than 26 would not converge due to a low number of transitions of disability recovery in those who were cognitively impaired. Cancer was excluded from the list of LTCs as there was no data for cancer in CFAS I, and, therefore, a comparison could not be performed. The measure for disability was also limited to one available in both studies; however, other measures of disability or dependency may result in different estimates and trends in LE and DFLE [40]. Although becoming more common among studies analysing temporal comparisons, participation rate for baseline interviews decreased between CFAS I and CFAS II. Nonresponse was associated with similar factors in both studies [41], and we used inverse probability weighting to ensure population representativeness. Even so, the areas included in CFAS were majority white for the generations studied at the time, and, as we did not have information on race for those who did not participate in the study, this could not be accounted for in the weights. Therefore, these results cannot be seen as representative of ethnic minority communities. Finally, although CFAS I included many other follow-up interviews after 2 years, analysis had to be restricted to remain comparable with CFAS II, which only has a 2-year follow-up interview and 4.5 years vitals follow-up.

### Implications for policy and practice

Our study observed improvements to DFLE in the presence of most of the health conditions we included. Improvements in DFLE for people with stroke could be from decreases in stroke severity, potentially from increased use of preventive medicines or earlier treatment [42]. For people with diabetes, improved DFLE may be from lifestyle interventions such as weight loss and physical activity [43]. Healthier lifestyles and improved access to treatment may also have contributed to the improvement in CHD DFLE [44]. Further gains could be made through earlier diagnosis and greater access to beneficial treatments, although delays in screening, diagnosis, and treatment resulting from the Coronavirus Disease 2019 (COVID-19) pandemic may have a detrimental impact. Moreover, the negative association of cognitive impairment and DFLE for both men and women has significant implications for policy and clinical practice. Worldwide, dementia is already one of the most costly LTCs for the person with the illness, their family, and our wider society. Care costs are estimated to exceed 1 trillion dollars by 2030 [45]. In England and Wales [32,33], considerable geographical inequalities in postdiagnostic dementia care persist, which have been aggravated during the COVID-19 pandemic [34,35]. A similar situation exists in Europe [46]. The 2020 European Dementia Monitor Report compared change in dementia policy and care across 36 countries in and external to the European Union and showed that there are still insufficient postdiagnostic dementia support and care services especially for those with moderate dementia, although half of the participating countries reported an increase since 2017 [46]. With economic modelling predicting the number of people with dementia in England will more than double in the next 25 years, leading to a trebling of expenditure on dementia care [47], there is an urgent need to ensure that all people with dementia have access to evidence-based, high-quality care to enable them to live independently for as long as possible [48]. In addition, recent analysis estimates that 40% of dementia may be preventable through attention to 12 modifiable risk factors in earlier life [49]. Implementing interventions to address 3 of these (hypertension, smoking, and hearing loss) could improve health-related quality of life and reduce annual dementia care costs by £1.86 billion [49]. Furthermore, another modifiable risk factor for dementia, obesity, is also a leading risk factor for other LTCs we considered and could therefore be a target for strategies to prevent a substantial proportion of arthritis, diabetes, stroke, and CHD, which, in turn, could improve DFLE.

## Conclusions

Our study is the first, to our knowledge, to estimate temporal trends in LE and DFLE with health conditions from longitudinal data and separately for men and women. We found that the underlying transitions influencing trends in DFLE for those with health conditions differed between men and women. Improvements for women with health conditions may be related to reduced disability incidence and improvements for men from reductions in the probability of death. For women, reductions in incident disability were great enough that DFLE increased and DLE decreased in the presence of CHD. While these findings are positive, we also found a decline in the percentage of remaining years spent disability-free for men and women with cognitive impairment. Given that cognitive impairment was also the only LTC where prevalence decreased, this is a cause for concern and requires further investigation.

## Supporting information

S1 Text Prospective analysis plan, details of the analysis plan from the funding application. (DOCX)

S2 Text Questionnaire items used to determine variables in the analysis. (DOCX)

S3 Text Additional information on the disability-free life expectancy methods and inverse probability weights. (DOCX)

S4 Text Strengthening the Reporting of Observational Studies in Epidemiology (STROBE) guideline checklist. (DOCX)

S5 Text Supplementary results, additional tables reporting results from figures with confidence intervals and *p*-values, include the following:
Table A: Weighted prevalence (%) of long-term conditions by age in the Cognitive Function and Ageing Studies (CFAS I and CFAS II). Weighted prevalence of health conditions from CFAS I and CFAS II age and sex standardised to CFAS I population (1991) with 95% confidence intervals (95% CI).Table B: Prevalence of at least one other long-term condition in people with each specific long-term condition, by sex and study—Cognitive Function and Ageing Studies (CFAS I and CFAS II).Table C: Life expectancy (LE), disability-free life expectancy (DFLE), and life expectancy with disability (DLE) with 95% confidence intervals (95% CI) at age 65 for men with and without long-term conditions in the first and second Cognitive Function and Ageing Studies (CFAS I and CFAS II).Table D: Percentage of remaining years at age 65 spent disability-free (DFLE %) or with disability (DLE %) for men with and without long-term conditions in the Cognitive Function and Ageing Studies (CFAS I and CFAS II).Table E: Life expectancy (LE), disability-free life expectancy (DFLE), and life expectancy with disability (DLE) with 95% confidence intervals (95% CI) at age 65 for women with and without long-term conditions in the Cognitive Function and Ageing Studies (CFAS I and CFAS II).Table F: Percentage of remaining years at age 65 spent disability-free (DFLE %) or with disability (DLE %) for women with and without long-term conditions in the Cognitive Function and Ageing Studies (CFAS I and CFAS II).Table G: Relative Risk Ratios (RRR) for transition with each long-term condition (relative to without condition) from unadjusted models for men in the Cognitive Function and Ageing Studies (CFAS I and CFAS II), with 95% confidence intervals (95% CI).Table H: Relative Risk Ratios (RRR) for transition with each long-term condition (relative to without condition) from unadjusted models for women in the Cognitive Function and Ageing Studies (CFAS I and CFAS II), with 95% confidence intervals (95% CI).
(DOCX)

## Abbreviations

ADL: activities of daily living
CFAS: Cognitive Function and Ageing Study
CHD: coronary heart disease
CI: confidence interval
COVID-19: Coronavirus Disease 2019
CVD: cardiovascular disease
DALY: disability-adjusted life year
DFLE: disability-free life expectancy
DLE: life expectancy with disability
IMaCh: Interpolated Markov Chain
LE: life expectancy
LTC: long-term condition
MLTC: multiple long-term condition
MMSE: Mini Mental State Examination
ONS: Office for National Statistics
OR: odds ratio
PVD: peripheral vascular disease
RRR: relative risk ratio
YLD: years lived with disability
YLL: years of life lost
