# Supplementary material for: A comparison over 2 decades of disability-free life expectancy at age 65 years for those with long-term conditions in England: Analysis of the 2 longitudinal Cognitive Function and Ageing Studies
Source: PLoS Med. 2022 Mar 15;19(3):e1003936. doi: 10.1371/journal.pmed.1003936 (PMC8923437; doi:10.1371/journal.pmed.1003936)
Supplement: S1 Text — (DOCX) [file pmed.1003936.s001.docx]

**S1 Text – Prospective analysis plan**

**Aims and objectives of project**

**What research questions are you seeking to answer?**

Aims

The overarching aim of this project is to better understand how health and longevity are co-evolving in order to maximise health life expectancy for all older people.

The project will answer the following research questions:

1. Are the extra years with disability and dependency experienced by older adults over the last 20 years due to (a) increased incidence of disability/dependency, (b) reduced ability to return to independence, or (c) longer survival with disability/dependency?

2. Are the extra years with disability due to individual long-term conditions becoming more prevalent, or more disabling, or because multiple concurrent conditions (multi-morbidity) have increased?

3. Are all social groups, defined by level of education (early life disadvantage) or social class (mid-life social disadvantage), experiencing similar trends, and underlying dynamics, in disability and dependency?

**Please describe the methods you are intending to use for your study (what you plan to do) and why you chose these methods.**

**Present all usual elements that are pertinent to your chosen methods including analysis, so that the reviewers can judge whether the study is feasible and properly planned. Given the priorities of the Trust, make reference to the relevant gerontological science and practice. Please note that figures and/or tables cannot be included in this answer but can be uploaded if you are invited to full application.**

**Please note that the answer you previously provided to this question has been pulled through from your outline application. It is expected that you will now wish to provide more detail on your protocol and methods.**

Data and measures

We will use the unique datasets, the Cognitive Function and Ageing Studies (CFAS I and II), two identical longitudinal studies of older adults aged 65 years and over (including those in residential care) conducted approximately 20 years apart in 1991 and 2011 in three geographical centres (Cambridge, Newcastle and Nottingham). General practices in the three areas provided lists of patients aged 65 years and over and these were stratified by age (equal samples of those aged 65-74 years and 75+ years in each centre). Participants were interviewed at home by trained interviewers. CFAS is the only dataset to be able to explore these trends longitudinally; national routine datasets such as the General Practice Research Database, do not contain information on disability and the English Longitudinal Study of Ageing only includes current cohorts of older adults and not the previous generation.

As we are undertaking more in-depth analyses of previously reported trends in

disability and dependency (Jagger et al., 2016, Kingston et al., 2017), we shall utilise the same measures. Disability will be defined based upon the hierarchy of basic activities of daily living (BADL) and Instrumental Activities of Daily Living (IADL) (Katz et al., 1963, Lawton and Brody, 1969), and classifying participants into one of three levels: no disability, mild disability (requires help with IADL only), moderate or severe disability (requires help with BADL). Dependency will be defined using the interval of need (Isaacs and Neville, 1976) and based on IADL, BADL, incontinence and cognitive impairment; individuals are classified on the basis of the lapsed time between periods when they might require help into four categories: independent (supervision or help not essential), low dependency (requiring help less often than daily), medium dependency (requiring help at regular intervals each day), and high

dependency (requiring 24-h care, because help needed at any time, or constant supervision needed).

The long-term conditions to be investigated for their impact on disability will be

predominantly those previously investigated in CFAS I (Jagger et al., 2007), and are mostly self-report of doctor diagnoses including: coronary heart disease, stroke, diabetes, chronic airways obstruction, and arthritis. Two further conditions are interviewer observed (visual impairment, hearing impairment), and another two derived from diagnostic scales: peripheral vascular disease (Rose et al., 1977), and cognitive impairment (Folstein et al., 1975). We shall also investigate the co-occurrence of long-term conditions in two ways. Firstly as a simple summation of the number of long-term conditions to reflect multi-morbidity (2+ conditions) and complex multi-morbidity (4+ conditions) (Kingston et al., 2018). Secondly specific combinations of conditions that have been identified as particularly problematic for older people and their carers through the James Lind Alliance Priority Setting Partnership on multiple conditions in later life which will report in April 2018.

All participants were flagged on the UK Office of National Statistics National Health Service Central Register for notification of date of death.

Analytic methods

The previous trend analyses have used cross-sectional data and Sullivan’s method (Sullivan, 1971) to calculate DFLE and DepFLE. Multi-state life table techniques with longitudinal data enable estimation of the underlying dynamic processes of the health expectancies. The project team (Jagger, Matthews, Kingston) have expertise in all major software packages for analysis of healthy life expectancy from longitudinal data and have published widely with them. We anticipate analyses will use the Interpolated Markov Chain (IMaCH) package developed at the National Institute of Demography in Paris (Lievre et al., 2003). This technique partitions the time between successive interviews into shorter steps and then models the resulting transition probabilities by multinomial logistic regression on age (and other covariates). Estimated transition probabilities then act as inputs to a multistate life table. We will run a series of models for each of the measures of disability and dependency; men and women will be modelled separately.

To answer the first research question we will run separate models for each time period/study and compare the overall conclusion drawn on the previous time trends of DFLE and DepFLE from cross-sectional data and the longitudinal estimates produced by the project. We expect the estimates may differ a little as the cross-sectional ones utilise national period life expectancy whilst the longitudinal estimates utilise the study-specific mortality. However overall conclusions on time trends are unlikely to differ. Estimates of the transition probabilities between states corresponding to incidence, recovery and state-specific mortality, will be compared between the two time points by

comparing the difference over time to its variance (sum of variance of estimate for each time point). This assumes that the estimates from the two time points are independent which is likely as there is little overlap in participants across the twenty-year time period between the two studies.

For the second research question binary variables for the presence/absence of each long-term condition at baseline will be created and each used in turn in a model to assess the impact of each condition on transitions to and from disability and death at each time point. The difference in life expectancy (LE), DFLE and years with disability (DLE) between participants with, and those without, the disease will provide estimates of the disabling effect and fatality of each condition and these will be compared over time as for the previous research question. Models will be repeated with multi-morbidity as a covariate (defined as: 0,1,2+ conditions; 0-1, 2-3, 4+ conditions), and with the presence of specific key conditions as outlined earlier. To achieve convergence we may have to collapse categories of disability and run models with disability defined as none/mild+ and none+mild/severe for some conditions.

Finally the third research questions examines whether inequalities in DFLE or DepFLE have increased over time. Here we will use different measures of social advantage (education or social class) as covariates and estimate the difference in LE, DFLE and DLE between the highest and lowest category of social advantage, comparing these across the two time points. This again may require collapsing of categories to achieve convergence, especially for models with dependency.
