# Supplementary material for: A comparison over 2 decades of disability-free life expectancy at age 65 years for those with long-term conditions in England: Analysis of the 2 longitudinal Cognitive Function and Ageing Studies
Source: PLoS Med. 2022 Mar 15;19(3):e1003936. doi: 10.1371/journal.pmed.1003936 (PMC8923437; doi:10.1371/journal.pmed.1003936)
Supplement: S2 Text — (DOCX) [file pmed.1003936.s002.docx]

**S2 Text – Questionnaire items**

Question numbers all from the first Cognitive Function and Ageing (CFAS I) questionnaire but questions are the same in CFAS II.

**Disability**

| Q122 Are you able to wash all over or bath? (If YES: Do you have difficulty?)  0. No, needs help  1. Yes, some difficulty  2. Yes, no difficulty  7. Don’t know  8. No answer  9. Not asked | People with mental frailties who cannot undertake activities because of their mental frailty should be coded as needing help. |
| --- | --- |
| Q125 Are you able to do heavy housework? (If YES: Do you have difficulty?)  0. No, needs help  1. Yes, some difficulty  2. Yes, no difficulty  7. Don’t know  8. No answer  9. Not asked | Q125 Heavy Housework – for example, washing floors. |
| Q126 Are you able to shop and carry heavy bags? (If YES: Do you have difficulty?  0. No, needs help  1. Yes, some difficulty  2. Yes, no difficulty  7. Don’t know  8. No answer  9. Not asked |  |
| Q127 Are you able to prepare and cook a hot meal? (If YES: Do you have difficulty?)  0. No, needs help  1. Yes, some difficulty  2. Yes, no difficulty  7. Don’t know  8. No answer  9. Not asked | Q127 If the subject claims they never have to cook a hot meal because this is always done for them, ask them to make a judgement as to whether they could if they had to. |
| Q130 Are you able to put on your shoes and socks or stockings? (If YES: do you have difficulty?)  0. (No), needs help  1. (Yes), some difficulty  2. (Yes), no difficulty  7. Don’t know  8. No answer  9. Not asked |  |
| ITEM 149 Establish degree of mobility of subject.  1. Usually ambulant non-housebound  2. Usually ambulant housebound  3. Chairfast permanently  4. Bedfast permanently  7. Unable to establish mobility |  |

**Health conditions**

| **I'm now going to ask about your general health.** |  |  |
| --- | --- | --- |
| Q41 Have you ever suffered with angina.  0. No  1. Yes  8. No answer  9. Not asked |  |  |
| Q51 Have you ever suffered from intermittent claudication?  0. No  1. Yes  8. No answer  9. Not asked | Q61 Have you ever suffered from a heart attack?  0. No  1. Yes  8. No answer  9. Not asked | |
| Q69 Have you ever had a stroke that required medical attention?  0. No  1. Yes  8. No answer  9. Not asked | Q77 Have you ever had sugar diabetes?  0. No  1. Yes  8. No answer  9. Not asked | |
| Q95 Do you suffer from hearing problems which interfere with day-to-day living?  0. No  1. Yes  8. No answer  9. Not asked | ITEM 202 Did the subject have hearing problems that interfered with the questioning?  0. No  1. To some extent  2. To a marked extent  3. Deaf  9. Inapplicable |  |
| Q96 Do you suffer from poor eyesight which interferes with day-to-day living?  0. No  1. Yes  8. No answer  9. Not asked | ITEM 200 Did the subject have poor/no eyesight that interfered with reading, writing or drawing?  0. No  1. To some extent  2. To a marked extent  3. Blind  9. Inapplicable |  |
| Q97 Have you ever suffered from asthma?  0. No  1. Yes, childhood only  2. Yes  8. No answer  9. Not asked |  |  |
| Q98 Have you ever suffered from arthritis?  0. No  1. Yes  8. No answer  9. Not asked |  |  |
| Q99 Have you ever suffered with chronic bronchitis?  0. No  1. Yes  8. No answer  9. Not asked |  |  |

**Health conditions – Cognitive impairment (MMSE)**

| Q3 What is the name of this place? Where is it located.   1. Correct 2. Error in name 3. Error in address 4. Error in name and address 5. Don't know 6. No answer 7. Not asked | Q3 Rate 1-Correct, if subject gives only a partial name.  Rate 3-Error in address, if subject only gives locality. |
| --- | --- |
| Q4 What is the name of this city/town/village?   1. Incorrect 2. Correct 3. Don't know 4. No answer   Not asked | Q4 A localising answer is required here. If the subject lives in a remote area, the name of the nearest farm would be considered correct.  The answer to this question will be scored either as part of Q3 or Q5 |
| ONLY ASK THIS QUESTION IF THE SUBJECT IS LIVING IN THEIR OWN HOME. OTHERWISE SKIP TO Q6. PROBE FOR FULL ADDRESS |  |
| Q5 What is the full address of this place?   1. Incorrect or incomplete 2. Correct 3. Don't know 4. No answer   Not asked | Q5 The full address is required. Probe for full address including city, suburb or postal district but not post code. If in a day hospital ask "What is the postal address of your home?" |
| *Q159 What day of the week is it today?  0. Incorrect  1. Correct  9. Inapplicable |  |
| *Q160 What is the date today?  Day  1. Correct  2. Incorrect by 1 day  3. Incorrect by >1 day  7. Don’t know  8. No answer  9. Not asked  Month  1. Correct  2. Incorrect by 1 month  3. Incorrect by >1 month  7. Don’t know  8. No answer  9. Not asked  Year  1. Correct  2. Incorrect by 1 year  3. Incorrect by >1 year  7. Don’t know  8. No answer  9. Not asked | Rate as correct if the subject claims it is the previous month in the first week of the month (e.g. March in the first week of April). |
| *Q161 What is the season?  0. Incorrect  1. Correct  9. Inapplicable | Q161 Allow flexibility when season changes, e.g.:  March = winter/spring  June = spring/summer  September = summer/autumn  Late Nov/Dec = autumn/winter |
| **Can you tell me where we are now? For instance:** |  |
| *Q162 What county are we in?  0. Incorrect  1. Correct  9. Inapplicable |  |
| *Q163 Name two main streets nearby (or near to your home)?  0. Incorrect  1. Correct  9. Inapplicable | Q163 Not including their own street. |
| *Q164 What floor of this building are we on?  0. Incorrect  1. Correct  9. Inapplicable |  |
| SHOW PENCIL |  |
| *Q166 What is this called?  0. Incorrect  1. Correct  9. Inapplicable | Q166 – 169 For these questions accurate naming is required. Descriptions of function or approximate answers are not acceptable. For example: used for telling the time, for wristwatch, would be incorrect.  Present the objects to the subject and allow them to be held. Put the objects out of sight before proceeding. |
| SHOW WRISTWATCH |  |
| *Q167 What is this called?  0. Incorrect  1. Correct  9. Inapplicable |  |
| **I am now going to say something and I would like you to repeat it after me.** |  |
| *ITEM 171 No ifs, and or buts.  0. Incorrect  1. Correct  9. Inapplicable | I170 Only one presentation is allowed so it is essential that you read the phrase clearly and slowly, enunciating all the S’s. |
| *ITEM 178 I am now going to say three words. After I have finished saying all three, I want you to repeat them. Remember what they are because I am going to ask you to name them in a few minutes. NAME THESE 3 OBJECTS TAKING 1 SECOND TO SAY EACH:  Apple, Table, Penny  Apple  0. Not named on first try  1. Named on first try  9. Not asked  Table  0. Not named on first try  1. Named on first try  9. Not asked  Penny  0. Not named on first try  1. Named on first try  9. Not asked | If any errors or omissions are made on the first attempt, repeat all the names until subject learns all three up to a maximum of five repeats. |
| *ITEM 179 Now I would like you to take 7 away from 100. Now take 7 away from the number you get.  Now keep taking 7 away until I tell you to stop.  Record the answers:  nn, nn, nn, nn, nn.  888 No answer  999 Not asked | ITEM 179 Score 1 point for each time the difference is 7, even if a previous answer was incorrect. Maximum score 5 points. DO NOT repeat the number you were given. Enter 888 if No answer given and 999 for Not asked. An entry of 999 will cause a skip to the next question. |
| *Q180 What were the three words I asked you to repeat a little while ago?  Apple  0. Not recalled  1. Recalled  9. Not asked  Table  0. Not recalled  1. Recalled  9. Not asked  Penny  0. Not recalled  1. Recalled  9. Not asked |  |
| OFFER SUBJECT THE SENTENCE AT THE END OF THE HANDBOOK (Close your eyes) |  |
| ITEM 181 Please read what is here and do what it says.  0. Incorrect  1. Correct  9. Inapplicable | ITEM 181 It is not necessary for the subject to read aloud. Score as correct only if the action is correctly carried out. If the subject reads the instruction but fails to carry out the action, say ‘Now do what it says’. |
| OFFER SUBJECT THE DRAWING PAGE |  |
| *ITEM 182 Here is a drawing. Please copy the drawing on the same paper.  0. Incorrect  1. Correct  9. Inapplicable | ITEM 182 Correct if the two five-sided figures intersect to form a four-sided figure and if all the angles in the five-sided figures are preserved.  NB Copy to be below the original and drawing to be done freehand. If will not attempt it mark as incorrect. |
| *ITEM 183 Write a complete sentence on this sheet of paper.  0. Incorrect  1. Correct  9. Inapplicable | ITEM 183 Sentence to be written in the box on the same piece of paper as the previous item. Spelling and grammar are not important. The sentence must have a subject (real or implied) and a verb. ‘Help’ or ‘Go away’ are acceptable. If sentence is illegible ask subject what they have written and write it underneath. Under no circumstances should you write it for them from dictation. |
| RATE: *IS THE SUBJECT LEFT- OR RIGHT-HANDED?  1. Right-handed  2. Left-handed  3. Ambidextrous  7. Unable to judge |  |
| READ FULL STATEMENT STRESSING THE WORDS IN CAPITALS AND THEN HAND OVER PAPER |  |
| *ITEM 184 I am now going to give you a piece of paper. When I do, take the paper in your RIGHT hand. Fold the paper in HALF with BOTH hands and put the paper down on your LAP.  Takes in right hand  0. Incorrect  1. Correct  9. Inapplicable  Folds in half  0. Incorrect  1. Correct  9. Inapplicable  Places on lap  0. Incorrect  1. Correct  9. Inapplicable | ITEM 184 If the full sequence is not completed repeat the whole instruction to ensure that it has been heard and understood. Do not prompt.  Score a move as correct only if it takes place in the correct sequence. Modify instruction for one-handed subjects. If one-handed score “Takes in right hand” as 9. |
