# Supplementary material for: A comparison over 2 decades of disability-free life expectancy at age 65 years for those with long-term conditions in England: Analysis of the 2 longitudinal Cognitive Function and Ageing Studies
Source: PLoS Med. 2022 Mar 15;19(3):e1003936. doi: 10.1371/journal.pmed.1003936 (PMC8923437; doi:10.1371/journal.pmed.1003936)

**S3 Text – Statistical methods**

*Estimating life and health expectancies*

Markov chains describe transitions between different states over time where time is modelled discretely or continuously and the transitions between states are defined by transition probabilities. Here, a three state model was estimated using interpolated Markov chain modelling, in which time was modelled discretely. There were two non-absorbing states, disability-free (state 1) and with disability (state 2) and one absorbing state, death (state 3), the transitions between these states are shown in S3 Figure 1. Methods described here are from Lievre et al.[1]

**S3 Figure 1**: Transitions between disability states and death in the three state multistate models used to estimate life expectancy and disability-free life expectancy.


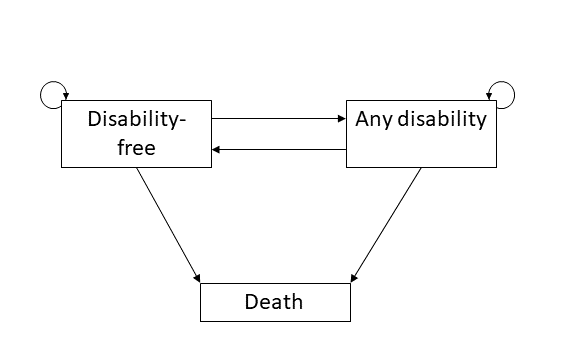


If $X(x)$ denotes the state of an individual aged $x$ and $X(x+m)$ is their state after time $m$ has elapsed, then the transition probability is given by

$${}_{m}{p_{x}^{ij}=\Pr\left( X\left( x+m \right)=j \right|X\left( x \right)=i)}$$

where ${{}_{m}p}_{x}^{ij}$ is the entry $(i, j)$ in the transition probability matrix

$${}_{m}{P_{x}}=\left( \begin{matrix} {{}_{m}p}_{x}^{11} & {}_{m}{p_{x}^{12}} & {}_{m}{p_{x}^{13}} \\ {}_{m}{p_{x}^{21}} & {}_{m}{p_{x}^{22}} & {}_{m}{p_{x}^{23}} \\ 0 & 0 & 1 \end{matrix} \right) .$$

The transition probability ${{}_{m}p}_{x}^{ij}$ can be parameterised through the multinomial logistic regression model

$$ln\left( \frac{{}_{m}{p_{x}^{ij}}}{{}_{m}{p_{x}^{ii}}} \right)= \alpha_{ij}\left( m \right)+ \beta_{ij}\left( m \right)x , i\neq j (1)$$

where $m$ is a fixed time step of $m= 1 month$ and ${}_{m}{p_{x}^{ii}}$ is the probability of remaining in state $i$ after time $m$ has elapsed.

Given that the gap between baseline and follow-up interview in the Cognitive Function and Ageing Studies (CFAS I and CFAS II) was two years, the probability of transitioning within those two years can be found from the product of transition probability matrices:

$$\prod_{u=1}^{n} {}_{m}{P_{x+\left( u-1 \right)m}}$$

where $n$ is the number of steps $m$ between interviews (in this case $n=24$). The same product of matrices can be used for vitals follow-up between interview and death, however $n$ would vary as exact date of death was known. Therefore, for any given time interval $\left( x, x+y \right)$ where $y=nm$, the transition probability ${}_{y}{p_{x}^{ij}}$ is the $(i, j)$ entry of the matrix product $\left( {}_{m}{P_{x})({}_{m}{P_{x+m}}} \right)({}_{m}{P_{x+2m})}\ldots({}_{m}{P_{y-m}})$. The individual’s contribution to the likelihood is then the product of transition probability matrices over the study period and the likelihood can be maximised to estimate the parameters $\alpha_{ij}\left( m \right)$ and $\beta_{ij}\left( m \right)$ from equation $(1)$.

The transition probabilities can directly be used to estimate health expectancies. The health expectancy $e_{x}^{ij}$, the expected time spent in state $j$ by an individual who was in state $i$ at age $x$ is given by

$$e_{x}^{ij}= \sum_{y=1}^{\infty} {}_{y}{p_{x}^{ij}} .$$

The probabilities ${}_{y}{p_{x}^{ij}}(\theta)$ are estimated by ${}_{y}{p_{x}^{ij}}(\hat{\boldsymbol{\theta}})$ where $\hat{\boldsymbol{\theta}}$ is the vector of maximum likelihood estimates for the parameters of equation $(1)$, such that the health expectancies can be estimated from

$$\hat{e_{x}^{ij}}= e_{x}^{ij}(\hat{\boldsymbol{\theta}}) = \sum_{y=1}^{\infty} {}_{y}{p_{x}^{ij}}(\hat{\boldsymbol{\theta}}) .$$

Weighted averages of $e_{x}^{1j}$ and $e_{x}^{2j}$ give the overall health expectancy of state $j$ regardless of the starting state and the weights are the proportion of the sample in state 1 ($\pi_{x}^{1}$) and state 2 ($\pi_{x}^{2}$) at age $x$, such that

$$e_{x}^{.j}= \pi_{x}^{1}e_{x}^{1j}+\pi_{x}^{2}e_{x}^{2j} .$$

As an example, disability-free life expectancy at age 65 would be given by

$$e_{65}^{.1}= \pi_{65}^{1}e_{65}^{11}+\pi_{65}^{2}e_{65}^{21} .$$

1. Lièvre, A., N. Brouard, and C. Heathcote, *The Estimation of Health Expectancies from Cross-Longitudinal Surveys.* Mathematical Population Studies, 2003. **10**(4): p. 211-248.

*Inverse probability weighting for life expectancies*

Inverse probability weighting was used for the life expectancy analysis to ensure population representativeness. Here the weights used for the life expectancy analysis are described. The weighting differs to the weighting used for the PAF models as those who died were included in the life expectancy models but not in the PAF models. Anyone who was still alive by the censor date and who participated in baseline and the two-year follow up interview (Group A, S3 Figure 2) were baseline weighted and also longitudinally weighted. The longitudinal weights compared Group A (S3 Figure 2) to Group B (S3 Figure 2) and were based on age, sex, centre, deprivation, education, social class, cognitive function, disability, number of long-term conditions (count), self-rated health and smoking. Anyone who was alive by the censor date but only participated in the baseline interview (Group B, S3 Figure 2) made no recorded transitions and were then excluded from the life expectancy models but accounted for in the longitudinal weights. Anyone who died before the censoring date (censoring date four years after baseline) (Group C, S3 Figure 2) were baseline weighted for age, sex, deprivation and care home status. Additionally, as CFAS I and CFAS II were healthy cohorts, a weight comparing probability of death in CFAS to ONS probability of death was applied. To create these weights probability of death at each year of age was estimated for CFAS I and CFAS II separately. Probability of death at each year of age in CFAS I was compared to the equivalent from the ONS in years 1991-1993. CFAS II probability of death was compared to ONS years 2008-2010. As an example, the additional weight for those who died at age 72 in CFAS II was [probability of death at age 72 from the ONS 2008-2010] divided by [probability of death at age 72 from CFAS II].

**S3 Figure 2:** Possible routes from baseline through study period for a participant (arrow head indicates death)


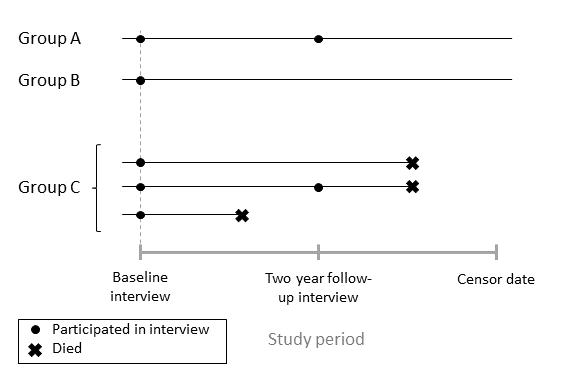

Supplement: S3 Text — (DOCX) [file pmed.1003936.s003.docx]
