# Supplementary material for: A comparison over 2 decades of disability-free life expectancy at age 65 years for those with long-term conditions in England: Analysis of the 2 longitudinal Cognitive Function and Ageing Studies
Source: PLoS Med. 2022 Mar 15;19(3):e1003936. doi: 10.1371/journal.pmed.1003936 (PMC8923437; doi:10.1371/journal.pmed.1003936)
Supplement: S5 Text — Supplementary results, additional tables reporting results from figures with confidence intervals and p-values, include the following: Table A: Weighted prevalence (%) of long-term conditions by age in the Cognitive Function and Ageing Studies (CFAS I and CFAS II). Weighted prevalence of health conditions from CFAS I and CFAS II age and sex standardised to CFAS I population (1991) with 95% confidence intervals (95% CI).Table B: Prevalence of at least one other long-term condition in people with each specific long-term condition, by sex and study—Cognitive Function and Ageing Studies (CFAS I and CFAS II).Table C: Life expectancy (LE), disability-free life expectancy (DFLE), and life expectancy with disability (DLE) with 95% confidence intervals (95% CI) at age 65 for men with and without long-term conditions in the first and second Cognitive Function and Ageing Studies (CFAS I and CFAS II).Table D: Percentage of remaining years at age 65 spent disability-free (DFLE %) or with disability (DLE %) for men with and without long-term conditions in the Cognitive Function and Ageing Studies (CFAS I and CFAS II).Table E: Life expectancy (LE), disability-free life expectancy (DFLE), and life expectancy with disability (DLE) with 95% confidence intervals (95% CI) at age 65 for women with and without long-term conditions in the Cognitive Function and Ageing Studies (CFAS I and CFAS II).Table F: Percentage of remaining years at age 65 spent disability-free (DFLE %) or with disability (DLE %) for women with and without long-term conditions in the Cognitive Function and Ageing Studies (CFAS I and CFAS II).Table G: Relative Risk Ratios (RRR) for transition with each long-term condition (relative to without condition) from unadjusted models for men in the Cognitive Function and Ageing Studies (CFAS I and CFAS II), with 95% confidence intervals (95% CI).Table H: Relative Risk Ratios (RRR) for transition with each long-term condition (relative to without condition) from unadjusted mod [file pmed.1003936.s005.docx]

**S5 Text – Supplementary results**

*Health condition prevalence results*

**Table A:** Weighted prevalence (%) of long-term conditions by age in the Cognitive Function and Ageing Studies (CFAS I and CFAS II). Weighted prevalence of health conditions from CFAS I and CFAS II age and sex standardised to CFAS I population (1991) with 95% confidence intervals (95% CI).

|  | **65-74** | | **75-84** | | **85+** | | **All ages** | | **All ages, standardised to 1991 population** | | | | | |
| --- | --- | --- | --- | --- | --- | --- | --- | --- | --- | --- | --- | --- | --- | --- |
|  | **CFAS I**  **%** | **CFAS II**  **%** | **CFAS I**  **%** | **CFAS II**  **%** | **CFAS I**  **%** | **CFAS II**  **%** | **CFAS I**  **%** | **CFAS II**  **%** | **CFAS I %** | **CFAS I 95% CI** | | **CFAS II %** | **CFAS II 95% CI** | |
| Arthritis | 50.0 | 50.1 | 55.3 | 57.1 | 57.0 | 64.2 | 52.9 | 55.0 | 52.4 | (48.8, | 56.0) | 54.6 | (51.0, | 58.1) |
| Cognitive impairment | 23.4 | 15.9 | 44.4 | 30.6 | 72.6 | 50.6 | 37.5 | 26.8 | 35.2 | (32.0, | 38.5) | 24.3 | (21.3, | 27.5) |
| Coronary Heart Disease | 15.7 | 16.3 | 19.7 | 24.9 | 19.7 | 26.3 | 17.7 | 21.0 | 17.4 | (14.8, | 20.3) | 19.7 | (16.9, | 22.8) |
| Diabetes | 5.3 | 14.1 | 7.6 | 16.1 | 5.5 | 11.6 | 6.2 | 14.5 | 6.1 | (4.6, | 8.2) | 14.3 | (11.9, | 17.1) |
| Hearing difficulties | 15.2 | 19.6 | 24.4 | 28.6 | 45.4 | 43.5 | 22.5 | 26.9 | 21.2 | (18.4, | 24.2) | 24.3 | (21.3, | 27.6) |
| Peripheral Vascular Disease | 4.0 | 10.2 | 4.6 | 11.3 | 4.0 | 10.8 | 4.3 | 10.7 | 4.2 | (3.0, | 6.0) | 10.6 | (8.5, | 13.1) |
| Respiratory problems | 20.0 | 19.8 | 18.3 | 20.5 | 19.2 | 16.4 | 19.2 | 19.5 | 19.3 | (16.6, | 22.4) | 20.0 | (17.2, | 23.1) |
| Stroke | 5.6 | 6.2 | 10.1 | 10.4 | 10.6 | 13.2 | 8.0 | 8.9 | 7.6 | (5.9, | 9.8) | 8.1 | (6.2, | 10.4) |
| Vision impairment | 7.1 | 11.4 | 15.9 | 15.1 | 32.7 | 26.8 | 13.6 | 15.2 | 12.5 | (10.3, | 15.0) | 14.1 | (11.7, | 16.9) |

**Table B:** Prevalence of at least one other long-term condition in people with each specific long-term condition, by sex and study – Cognitive Function and Ageing Studies (CFAS I and CFAS II).

|  | **Men** | | **Women** | |
| --- | --- | --- | --- | --- |
|  | **CFAS I** | **CFAS II** | **CFAS I** | **CFAS II** |
| Arthritis | 72.5 | 79.6 | 73.6 | 75.7 |
| Cognitive impairment | 81.1 | 87.5 | 83.6 | 88.8 |
| Coronary Heart Disease | 85.1 | 87.5 | 92.0 | 93.3 |
| Diabetes | 84.9 | 86.9 | 91.0 | 91.2 |
| Hearing difficulties | 87.7 | 85.1 | 92.5 | 91.3 |
| Peripheral Vascular Disease | 88.7 | 92.8 | 94.2 | 96.6 |
| Respiratory problems | 83.0 | 87.2 | 88.4 | 90.2 |
| Stroke | 90.1 | 89.8 | 91.8 | 95.5 |
| Vision impairment | 92.4 | 89.1 | 94.3 | 91.8 |

*Life expectancy results*

**Table C**: Life expectancy (LE), Disability-free life expectancy (DFLE) and life expectancy with disability (DLE) with 95% confidence intervals (95% CI) at age 65 for men with and without long-term conditions in the first and second Cognitive Function and Ageing Studies (CFAS I and CFAS II). Difference (Diff.) between those with and without the health condition given with 95% CI and p-values (Diff. p).

|  |  | **CFAS I** | | | | | | | | | **CFAS II** | | | | | | | | |
| --- | --- | --- | --- | --- | --- | --- | --- | --- | --- | --- | --- | --- | --- | --- | --- | --- | --- | --- | --- |
|  |  | **LE** | **LE 95% CI** | | **DFLE** | **DFLE 95% CI** | | **DLE** | **DLE 95% CI** | | **LE** | **LE 95% CI** | | **DFLE** | **DFLE 95% CI** | | **DLE** | **DLE 95% CI** | |
| Arthritis | No | 14.1 | (13.3, | 14.9) | 10.9 | (10.0, | 11.8) | 3.2 | (2.7, | 3.6) | 18.0 | (17.2, | 18.9) | 14.7 | (13.7, | 15.6) | 3.4 | (2.9, | 3.8) |
|  | Yes | 14.2 | (13.3, | 15.1) | 10.2 | (9.2, | 11.1) | 4.0 | (3.4, | 4.6) | 17.3 | (16.3, | 18.2) | 12.3 | (11.3, | 13.3) | 5.0 | (4.3, | 5.7) |
|  | Diff. | -0.1 | (-1.3, | 1.1) | 0.7 | (-0.6, | 2.0) | -0.8 | (-1.5, | -0.1) | 0.8 | (-0.5, | 2.0) | 2.4 | (1.0, | 3.8) | -1.6 | (-2.4, | -0.8) |
|  | Diff. p |  |  | 0.87 |  |  | 0.29 |  |  | 0.03 |  |  | 0.21 |  |  | <0.001 |  |  | <0.001 |
| Cognitive impairment | No | 14.8 | (14.1, | 15.5) | 11.2 | (10.5, | 12.0) | 3.6 | (3.1, | 4.0) | 18.6 | (17.8, | 19.3) | 14.6 | (13.8, | 15.4) | 4.0 | (3.5, | 4.4) |
|  | Yes | 12.0 | (10.9, | 13.1) | 8.4 | (7.2, | 9.6) | 3.6 | (2.9, | 4.3) | 14.8 | (13.5, | 16.0) | 9.8 | (8.1, | 11.4) | 5.0 | (4.1, | 5.9) |
|  | Diff. | 2.8 | (1.5, | 4.1) | 2.8 | (1.4, | 4.3) | -0.1 | (-0.9, | 0.8) | 3.8 | (2.3, | 5.3) | 4.8 | (3.0, | 6.6) | -1.0 | (-2.0, | 0.0) |
|  | Diff. p |  |  | <0.001 |  |  | <0.001 |  |  | 0.82 |  |  | <0.001 |  |  | <0.001 |  |  | 0.05 |
| Coronary | No | 14.7 | (13.9, | 15.4) | 11.1 | (10.2, | 11.9) | 3.6 | (3.2, | 4.0) | 18.1 | (17.4, | 18.8) | 14.1 | (13.3, | 15.0) | 4.0 | (3.5, | 4.4) |
| Heart | Yes | 12.1 | (11.0, | 13.1) | 8.7 | (7.6, | 9.7) | 3.4 | (2.7, | 4.0) | 16.5 | (15.4, | 17.7) | 12.0 | (10.8, | 13.3) | 4.5 | (3.7, | 5.3) |
| Disease | Diff. | 2.6 | (1.3, | 3.9) | 2.4 | (1.0, | 3.7) | 0.2 | (-0.6, | 1.0) | 1.6 | (0.2, | 2.9) | 2.1 | (0.6, | 3.6) | -0.5 | (-1.4, | 0.4) |
|  | Diff. p |  |  | <0.001 |  |  | <0.001 |  |  | 0.62 |  |  | 0.02 |  |  | 0.006 |  |  | 0.28 |
| Diabetes | No | 14.3 | (13.6, | 15.0) | 10.8 | (10.0, | 11.5) | 3.6 | (3.2, | 3.9) | 18.3 | (17.6, | 19.0) | 14.1 | (13.3, | 14.9) | 4.2 | (3.7, | 4.6) |
|  | Yes | 11.5 | (9.8, | 13.3) | 8.3 | (6.5, | 10.1) | 3.2 | (2.1, | 4.4) | 15.5 | (14.2, | 16.8) | 11.7 | (10.3, | 13.0) | 3.8 | (3.0, | 4.6) |
|  | Diff. | 2.8 | (0.9, | 4.6) | 2.4 | (0.5, | 4.4) | 0.3 | (-0.9, | 1.5) | 2.8 | (1.3, | 4.2) | 2.4 | (0.8, | 4.0) | 0.4 | (-0.6, | 1.3) |
|  | Diff. p |  |  | 0.003 |  |  | 0.02 |  |  | 0.62 |  |  | <0.001 |  |  | 0.003 |  |  | 0.41 |
| Hearing difficulties | No | 14.3 | (13.5, | 15.0) | 10.7 | (10.0, | 11.5) | 3.5 | (3.1, | 3.9) | 18.0 | (17.3, | 18.8) | 13.9 | (13.1, | 14.8) | 4.1 | (3.6, | 4.6) |
|  | Yes | 13.6 | (12.4, | 14.8) | 10.0 | (8.7, | 11.3) | 3.6 | (3.0, | 4.3) | 17.2 | (16.1, | 18.3) | 12.9 | (11.5, | 14.2) | 4.4 | (3.7, | 5.0) |
|  | Diff. | 0.7 | (-0.7, | 2.0) | 0.8 | (-0.7, | 2.3) | -0.1 | (-0.9, | 0.7) | 0.8 | (-0.5, | 2.1) | 1.1 | (-0.5, | 2.7) | -0.3 | (-1.1, | 0.5) |
|  | Diff. p |  |  | 0.31 |  |  | 0.30 |  |  | 0.81 |  |  | 0.23 |  |  | 0.18 |  |  | 0.46 |
| Peripheral  Vascular | No | 14.2 | (13.6, | 14.9) | 10.7 | (10.0, | 11.5) | 3.5 | (3.1, | 3.9) | 18.0 | (17.3, | 18.7) | 13.9 | (13.1, | 14.7) | 4.1 | (3.7, | 4.5) |
|  | Yes | 12.1 | (10.1, | 14.1) | 8.3 | (6.3, | 10.2) | 3.8 | (2.5, | 5.2) | 15.6 | (13.9, | 17.3) | 11.6 | (10.0, | 13.2) | 4.1 | (2.9, | 5.2) |
| Disease | Diff. | 2.1 | (0.1, | 4.2) | 2.4 | (0.4, | 4.5) | -0.3 | (-1.7, | 1.1) | 2.3 | (0.5, | 4.2) | 2.3 | (0.5, | 4.1) | 0.0 | (-1.2, | 1.3) |
|  | Diff. p |  |  | 0.04 |  |  | 0.02 |  |  | 0.67 |  |  | 0.01 |  |  | 0.01 |  |  | 0.96 |
| Respiratory | No | 14.9 | (14.2, | 15.6) | 11.2 | (10.5, | 12.0) | 3.7 | (3.2, | 4.1) | 18.0 | (17.2, | 18.7) | 13.8 | (13.0, | 14.6) | 4.2 | (3.7, | 4.6) |
| difficulties | Yes | 11.5 | (10.4, | 12.6) | 8.2 | (7.0, | 9.4) | 3.3 | (2.6, | 3.9) | 16.8 | (15.5, | 18.1) | 12.7 | (11.2, | 14.3) | 4.0 | (3.2, | 4.8) |
|  | Diff. | 3.4 | (2.1, | 4.7) | 3.0 | (1.6, | 4.4) | 0.4 | (-0.4, | 1.2) | 1.2 | (-0.3, | 2.7) | 1.0 | (-0.7, | 2.8) | 0.1 | (-0.8, | 1.1) |
|  | Diff. p |  |  | <0.001 |  |  | <0.001 |  |  | 0.33 |  |  | 0.12 |  |  | 0.26 |  |  | 0.84 |
| Stroke | No | 14.7 | (14.0, | 15.4) | 11.2 | (10.5, | 11.9) | 3.5 | (3.1, | 3.9) | 18.1 | (17.4, | 18.7) | 14.1 | (13.3, | 14.8) | 4.0 | (3.6, | 4.4) |
|  | Yes | 9.5 | (8.1, | 10.9) | 5.2 | (3.6, | 6.7) | 4.3 | (3.3, | 5.4) | 14.9 | (13.2, | 16.6) | 9.4 | (7.4, | 11.5) | 5.4 | (4.2, | 6.7) |
|  | Diff. | 5.2 | (3.6, | 6.7) | 6.0 | (4.4, | 7.7) | -0.9 | (-2.0, | 0.2) | 3.2 | (1.3, | 5.0) | 4.6 | (2.5, | 6.8) | -1.5 | (-2.8, | -0.2) |
|  | Diff. p |  |  | <0.001 |  |  | <0.001 |  |  | 0.11 |  |  | <0.001 |  |  | <0.001 |  |  | 0.02 |
| Vision impairment | No | 14.3 | (13.6, | 15.0) | 10.8 | (10.0, | 11.5) | 3.5 | (3.1, | 3.9) | 17.9 | (17.2, | 18.6) | 13.9 | (13.1, | 14.7) | 4.0 | (3.6, | 4.4) |
|  | Yes | 12.2 | (10.5, | 13.8) | 8.1 | (6.3, | 9.9) | 4.1 | (3.0, | 5.1) | 16.7 | (15.1, | 18.3) | 11.7 | (9.8, | 13.6) | 5.0 | (4.1, | 6.0) |
|  | Diff. | 2.1 | (0.3, | 3.9) | 2.7 | (0.7, | 4.7) | -0.6 | (-1.7, | 0.5) | 1.1 | (-0.6, | 2.9) | 2.2 | (0.1, | 4.3) | -1.1 | (-2.1, | 0.0) |
|  | Diff. p |  |  | 0.02 |  |  | 0.008 |  |  | 0.29 |  |  | 0.22 |  |  | 0.04 |  |  | 0.04 |

**Table D**: Percentage of remaining years at age 65 spent disability-free (DFLE %) or with disability (DLE %) for men with and without long-term conditions in the Cognitive Function and Ageing Studies (CFAS I and CFAS II).

|  |  | **CFAS I** | | | | | | **CFAS II** | | | | | |
| --- | --- | --- | --- | --- | --- | --- | --- | --- | --- | --- | --- | --- | --- |
|  |  | **DFLE %** | **DFLE % 95% CI** | | **DLE %** | **DLE % 95% CI** | | **DFLE %** | **DFLE % 95% CI** | | **DLE %** | **DLE % 95% CI** | |
| Arthritis | No | 77.5 | (75.5, | 79.4) | 22.5 | (20.6, | 24.5) | 81.3 | (79.6, | 83.1) | 18.7 | (16.9, | 20.4) |
|  | Yes | 71.8 | (69.4, | 74.2) | 28.2 | (25.8, | 30.6) | 71.0 | (68.8, | 73.3) | 29.0 | (26.7, | 31.2) |
| Cognitive impairment | No | 76.0 | (74.2, | 77.8) | 24.0 | (22.2, | 25.8) | 78.5 | (77.0, | 80.0) | 21.5 | (20.0, | 23.0) |
|  | Yes | 69.9 | (66.8, | 72.9) | 30.1 | (27.1, | 33.2) | 66.3 | (62.8, | 69.7) | 33.7 | (30.3, | 37.2) |
| Coronary | No | 75.5 | (73.7, | 77.2) | 24.5 | (22.8, | 26.3) | 78.0 | (76.4, | 79.6) | 22.0 | (20.4, | 23.6) |
| Heart Disease | Yes | 72.0 | (68.5, | 75.4) | 28.0 | (24.6, | 31.5) | 72.8 | (69.8, | 75.8) | 27.2 | (24.2, | 30.2) |
| Diabetes | No | 75.1 | (73.5, | 76.7) | 24.9 | (23.3, | 26.5) | 77.1 | (75.5, | 78.6) | 22.9 | (21.4, | 24.5) |
|  | Yes | 72.0 | (65.9, | 78.0) | 28.0 | (22.0, | 34.1) | 75.4 | (71.9, | 78.9) | 24.6 | (21.1, | 28.1) |
| Hearing difficulties | No | 75.3 | (73.6, | 77.1) | 24.7 | (22.9, | 26.4) | 77.4 | (75.8, | 79.0) | 22.6 | (21.0, | 24.2) |
|  | Yes | 73.2 | (70.0, | 76.5) | 26.8 | (23.5, | 30.0) | 74.7 | (72.0, | 77.4) | 25.3 | (22.6, | 28.0) |
| Peripheral  Vascular Disease | No | 75.3 | (73.7, | 76.9) | 24.7 | (23.1, | 26.3) | 77.3 | (75.8, | 78.7) | 22.7 | (21.3, | 24.2) |
|  | Yes | 68.4 | (61.3, | 75.4) | 31.6 | (24.6, | 38.7) | 74.1 | (69.7, | 78.4) | 25.9 | (21.6, | 30.3) |
| Respiratory difficulties | No | 75.5 | (73.8, | 77.2) | 24.5 | (22.8, | 26.2) | 76.8 | (75.2, | 78.3) | 23.2 | (21.7, | 24.8) |
|  | Yes | 71.7 | (68.1, | 75.3) | 28.3 | (24.7, | 31.9) | 76.0 | (72.5, | 79.5) | 24.0 | (20.5, | 27.5) |
| Stroke | No | 76.3 | (74.7, | 77.9) | 23.7 | (22.1, | 25.3) | 78.0 | (76.6, | 79.4) | 22.0 | (20.6, | 23.4) |
|  | Yes | 54.3 | (48.5, | 60.1) | 45.7 | (39.9, | 51.6) | 63.5 | (58.2, | 68.7) | 36.5 | (31.3, | 41.8) |
| Vision | No | 75.6 | (73.9, | 77.2) | 24.4 | (22.8, | 26.1) | 77.7 | (76.3, | 79.2) | 22.3 | (20.8, | 23.7) |
| impairment | Yes | 66.5 | (61.3, | 71.8) | 33.5 | (28.2, | 38.7) | 69.9 | (65.6, | 74.2) | 30.1 | (25.8, | 34.4) |

**Table E**: Life expectancy (LE), Disability-free life expectancy (DFLE) and life expectancy with disability (DLE) with 95% confidence intervals (95% CI) at age 65 for women with and without long-term conditions in the Cognitive Function and Ageing Studies (CFAS I and CFAS II). Difference (Diff.) between those with and without the health condition given with 95% CI and p-values (Diff. p).

|  |  | **CFAS I** | | | | | | | | | **CFAS II** | | | | | | | | |
| --- | --- | --- | --- | --- | --- | --- | --- | --- | --- | --- | --- | --- | --- | --- | --- | --- | --- | --- | --- |
|  |  | **LE** | **LE 95% CI** | | **DFLE** | **DFLE 95% CI** | | **DLE** | **DLE 95% CI** | | **LE** | **LE 95% CI** | | **DFLE** | **DFLE 95% CI** | | **DLE** | **DLE 95% CI** | |
| Arthritis | No | 16.9 | (16.0, | 17.8) | 10.7 | (9.6, | 11.7) | 6.2 | (5.6, | 6.9) | 20.3 | (19.3, | 21.4) | 13.5 | (12.3, | 14.7) | 6.8 | (6.0, | 7.7) |
|  | Yes | 18.5 | (17.7, | 19.3) | 9.1 | (8.3, | 9.8) | 9.4 | (8.7, | 10.1) | 20.4 | (19.5, | 21.2) | 11.1 | (10.2, | 12.1) | 9.2 | (8.4, | 10.0) |
|  | Diff. | -1.6 | (-2.8, | -0.4) | 1.6 | (0.3, | 2.8) | -3.2 | (-4.2, | -2.2) | 0.0 | (-1.4, | 1.3) | 2.4 | (0.8, | 3.9) | -2.4 | (-3.5, | -1.2) |
|  | Diff. p |  |  | 0.009 |  |  | 0.01 |  |  | <0.001 |  |  | 0.98 |  |  | 0.002 |  |  | <0.001 |
| Cognitive impairment | No | 19.0 | (18.2, | 19.8) | 10.4 | (9.7, | 11.1) | 8.6 | (7.9, | 9.3) | 21.1 | (20.3, | 21.9) | 13.0 | (12.1, | 13.8) | 8.2 | (7.5, | 8.8) |
|  | Yes | 16.2 | (15.2, | 17.2) | 8.5 | (7.5, | 9.4) | 7.7 | (6.9, | 8.6) | 18.0 | (16.7, | 19.4) | 8.7 | (7.3, | 10.1) | 9.3 | (8.1, | 10.6) |
|  | Diff. | 2.8 | (1.5, | 4.1) | 1.9 | (0.7, | 3.2) | 0.9 | (-0.3, | 2.0) | 3.1 | (1.5, | 4.6) | 4.2 | (2.6, | 5.9) | -1.2 | (-2.6, | 0.2) |
|  | Diff. p |  |  | <0.001 |  |  | 0.003 |  |  | 0.13 |  |  | <0.001 |  |  | <0.001 |  |  | 0.09 |
| Coronary | No | 17.9 | (17.3, | 18.6) | 10.2 | (9.5, | 10.9) | 7.8 | (7.2, | 8.3) | 20.4 | (19.7, | 21.2) | 12.3 | (11.5, | 13.2) | 8.1 | (7.5, | 8.8) |
| Heart | Yes | 16.0 | (14.1, | 17.9) | 6.7 | (5.6, | 7.9) | 9.3 | (7.7, | 10.9) | 18.0 | (15.8, | 20.2) | 9.4 | (7.8, | 11.1) | 8.5 | (6.9, | 10.2) |
| Disease | Diff. | 1.9 | (-0.1, | 3.9) | 3.4 | (2.1, | 4.8) | -1.5 | (-3.2, | 0.2) | 2.5 | (0.1, | 4.8) | 2.9 | (1.1, | 4.7) | -0.4 | (-2.2, | 1.4) |
|  | Diff. p |  |  | 0.06 |  |  | <0.001 |  |  | 0.08 |  |  | 0.04 |  |  | 0.002 |  |  | 0.66 |
| Diabetes | No | 18.0 | (17.3, | 18.6) | 10.1 | (9.4, | 10.7) | 7.9 | (7.4, | 8.5) | 20.6 | (19.8, | 21.3) | 12.5 | (11.7, | 13.3) | 8.1 | (7.4, | 8.7) |
|  | Yes | 14.9 | (12.7, | 17.2) | 5.6 | (3.8, | 7.4) | 9.3 | (7.1, | 11.6) | 17.8 | (15.9, | 19.7) | 8.7 | (7.1, | 10.4) | 9.1 | (7.4, | 10.8) |
|  | Diff. | 3.0 | (0.7, | 5.4) | 4.5 | (2.6, | 6.4) | -1.4 | (-3.7, | 0.9) | 2.7 | (0.7, | 4.8) | 3.8 | (1.9, | 5.6) | -1.0 | (-2.8, | 0.7) |
|  | Diff. p |  |  | 0.01 |  |  | <0.001 |  |  | 0.23 |  |  | 0.010 |  |  | <0.001 |  |  | 0.26 |
| Hearing difficulties | No | 17.7 | (17.0, | 18.4) | 9.9 | (9.2, | 10.5) | 7.9 | (7.3, | 8.5) | 20.4 | (19.7, | 21.2) | 12.2 | (11.4, | 13.0) | 8.2 | (7.6, | 8.9) |
|  | Yes | 18.0 | (16.6, | 19.4) | 9.4 | (8.0, | 10.7) | 8.6 | (7.4, | 9.8) | 19.4 | (17.9, | 21.0) | 11.2 | (9.6, | 12.7) | 8.3 | (7.0, | 9.5) |
|  | Diff. | -0.3 | (-1.8, | 1.3) | 0.5 | (-1.0, | 2.0) | -0.8 | (-2.1, | 0.6) | 1.0 | (-0.7, | 2.7) | 1.0 | (-0.7, | 2.8) | 0.0 | (-1.4, | 1.4) |
|  | Diff. p |  |  | 0.70 |  |  | 0.51 |  |  | 0.25 |  |  | 0.25 |  |  | 0.26 |  |  | 0.96 |
| Peripheral  Vascular | No | 17.8 | (17.2, | 18.5) | 9.9 | (9.2, | 10.5) | 8.0 | (7.4, | 8.5) | 20.2 | (19.5, | 20.9) | 12.3 | (11.5, | 13.0) | 8.0 | (7.3, | 8.6) |
|  | Yes | 17.9 | (14.2, | 21.6) | 7.7 | (5.5, | 9.8) | 10.2 | (7.0, | 13.4) | 20.0 | (17.4, | 22.6) | 9.5 | (7.4, | 11.6) | 10.5 | (8.2, | 12.7) |
| Disease | Diff. | 0.0 | (-3.8, | 3.7) | 2.2 | (-0.1, | 4.4) | -2.2 | (-5.5, | 1.1) | 0.2 | (-2.5, | 2.9) | 2.7 | (0.5, | 5.0) | -2.5 | (-4.8, | -0.2) |
|  | Diff. p |  |  | 0.99 |  |  | 0.06 |  |  | 0.19 |  |  | 0.88 |  |  | 0.02 |  |  | 0.03 |
| Respiratory difficulties | No | 18.0 | (17.3, | 18.7) | 10.3 | (9.6, | 11.0) | 7.7 | (7.1, | 8.3) | 20.7 | (19.9, | 21.5) | 12.7 | (11.9, | 13.5) | 8.0 | (7.4, | 8.6) |
|  | Yes | 17.0 | (15.7, | 18.3) | 8.0 | (6.9, | 9.1) | 9.0 | (7.9, | 10.2) | 18.5 | (17.1, | 20.0) | 9.8 | (8.4, | 11.2) | 8.7 | (7.4, | 10.0) |
|  | Diff. | 1.0 | (-0.5, | 2.5) | 2.3 | (1.0, | 3.6) | -1.3 | (-2.6, | 0.0) | 2.2 | (0.6, | 3.8) | 2.9 | (1.3, | 4.5) | -0.7 | (-2.1, | 0.7) |
|  | Diff. p |  |  | 0.19 |  |  | <0.001 |  |  | 0.05 |  |  | 0.007 |  |  | <0.001 |  |  | 0.33 |
| Stroke | No | 18.2 | (17.5, | 18.9) | 10.1 | (9.5, | 10.7) | 8.1 | (7.5, | 8.7) | 20.4 | (19.7, | 21.1) | 12.1 | (11.4, | 12.9) | 8.2 | (7.6, | 8.9) |
|  | Yes | 13.0 | (10.8, | 15.2) | 5.5 | (3.7, | 7.2) | 7.5 | (5.6, | 9.4) | 17.1 | (12.9, | 21.4) | 9.0 | (6.4, | 11.5) | 8.2 | (5.2, | 11.1) |
|  | Diff. | 5.2 | (2.9, | 7.5) | 4.6 | (2.8, | 6.5) | 0.6 | (-1.4, | 2.5) | 3.3 | (-1.0, | 7.5) | 3.2 | (0.5, | 5.9) | 0.1 | (-2.9, | 3.1) |
|  | Diff. p |  |  | <0.001 |  |  | <0.001 |  |  | 0.55 |  |  | 0.13 |  |  | 0.02 |  |  | 0.95 |
| Vision impairment | No | 17.8 | (17.1, | 18.5) | 9.9 | (9.3, | 10.6) | 7.9 | (7.3, | 8.5) | 20.5 | (19.7, | 21.2) | 12.4 | (11.5, | 13.2) | 8.1 | (7.5, | 8.8) |
|  | Yes | 16.2 | (14.1, | 18.3) | 7.8 | (6.5, | 9.2) | 8.4 | (6.7, | 10.1) | 18.7 | (16.9, | 20.5) | 9.9 | (8.2, | 11.6) | 8.8 | (7.3, | 10.3) |
|  | Diff. | 1.6 | (-0.6, | 3.8) | 2.1 | (0.6, | 3.6) | -0.5 | (-2.3, | 1.3) | 1.8 | (-0.1, | 3.7) | 2.5 | (0.6, | 4.3) | -0.7 | (-2.3, | 1.0) |
|  | Diff. p |  |  | 0.15 |  |  | 0.006 |  |  | 0.59 |  |  | 0.06 |  |  | 0.008 |  |  | 0.41 |

**Table F**: Percentage of remaining years at age 65 spent disability-free (DFLE %) or with disability (DLE %) for women with and without long-term conditions in the Cognitive Function and Ageing Studies (CFAS I and CFAS II).

|  |  | **CFAS I** | | | | | | **CFAS II** | | | | | |
| --- | --- | --- | --- | --- | --- | --- | --- | --- | --- | --- | --- | --- | --- |
|  |  | **DFLE %** | **DFLE % 95% CI** | | **DLE %** | **DLE % 95% CI** | | **DFLE %** | **DFLE % 95% CI** | | **DLE %** | **DLE % 95% CI** | |
| Arthritis | No | 63.1 | (60.9, | 65.2) | 36.9 | (34.8, | 39.1) | 66.3 | (64.0, | 68.7) | 33.7 | (31.3, | 36.0) |
|  | Yes | 49.1 | (47.2, | 51.0) | 50.9 | (49.0, | 52.8) | 54.7 | (52.8, | 56.6) | 45.3 | (43.4, | 47.2) |
| Cognitive impairment | No | 54.7 | (52.8, | 56.6) | 45.3 | (43.4, | 47.2) | 61.4 | (59.6, | 63.1) | 38.6 | (36.9, | 40.4) |
|  | Yes | 52.2 | (50.0, | 54.5) | 47.8 | (45.5, | 50.0) | 48.3 | (45.4, | 51.2) | 51.7 | (48.8, | 54.6) |
| Coronary | No | 56.7 | (55.1, | 58.3) | 43.3 | (41.7, | 44.9) | 60.3 | (58.7, | 62.0) | 39.7 | (38.0, | 41.3) |
| Heart Disease | Yes | 42.1 | (38.4, | 45.8) | 57.9 | (54.2, | 61.6) | 52.6 | (48.7, | 56.4) | 47.4 | (43.6, | 51.3) |
| Diabetes | No | 55.9 | (54.5, | 57.4) | 44.1 | (42.6, | 45.5) | 60.8 | (59.2, | 62.4) | 39.2 | (37.6, | 40.8) |
|  | Yes | 37.4 | (31.5, | 43.3) | 62.6 | (56.7, | 68.5) | 49.0 | (44.6, | 53.4) | 51.0 | (46.6, | 55.4) |
| Hearing difficulties | No | 55.6 | (54.0, | 57.2) | 44.4 | (42.8, | 46.0) | 59.7 | (58.0, | 61.4) | 40.3 | (38.6, | 42.0) |
|  | Yes | 52.1 | (48.9, | 55.2) | 47.9 | (44.8, | 51.1) | 57.4 | (54.3, | 60.5) | 42.6 | (39.5, | 45.7) |
| Peripheral  Vascular Disease | No | 55.2 | (53.8, | 56.7) | 44.8 | (43.3, | 46.2) | 60.6 | (59.1, | 62.2) | 39.3 | (37.8, | 40.9) |
|  | Yes | 42.9 | (35.0, | 50.8) | 57.1 | (49.2, | 65.0) | 47.7 | (42.7, | 52.6) | 52.3 | (47.4, | 57.3) |
| Respiratory difficulties | No | 57.0 | (55.4, | 58.6) | 43.0 | (41.4, | 44.6) | 61.3 | (59.6, | 63.0) | 38.7 | (37.0, | 40.4) |
|  | Yes | 46.8 | (43.5, | 50.2) | 53.2 | (49.8, | 56.5) | 53.0 | (49.7, | 56.3) | 47.0 | (43.7, | 50.3) |
| Stroke | No | 55.5 | (54.0, | 57.0) | 44.5 | (43.0, | 46.0) | 59.6 | (58.0, | 61.2) | 40.4 | (38.8, | 42.0) |
|  | Yes | 42.1 | (36.7, | 47.5) | 57.9 | (52.5, | 63.3) | 52.3 | (46.8, | 57.8) | 47.7 | (42.2, | 53.2) |
| Vision impairment | No | 55.8 | (54.2, | 57.3) | 44.2 | (42.7, | 45.8) | 60.3 | (58.7, | 61.9) | 39.7 | (38.1, | 41.3) |
|  | Yes | 48.4 | (44.7, | 52.1) | 51.6 | (47.9, | 55.3) | 52.9 | (49.1, | 56.7) | 47.1 | (43.3, | 50.9) |

**Table G:** Relative Risk Ratios (RRR) for transition with each long-term condition (relative to without condition) from unadjusted models for men in the Cognitive Function and Ageing Studies (CFAS I and CFAS II), with 95% confidence intervals (95% CI) and p-values (p).

|  |  | **CFAS I** | | | | **CFAS II** | | | |
| --- | --- | --- | --- | --- | --- | --- | --- | --- | --- |
|  |  | **RRR** | **95% CI** | | **p** | **RRR** | **95% CI** | | **p** |
| Arthritis | No disability -> Disability | 1.2 | (0.9, | 1.5) | 0.16 | 1.6 | (1.2, | 2.0) | <0.001 |
|  | No disability -> Death | 1.0 | (0.7, | 1.5) | 0.92 | 1.2 | (0.7, | 1.9) | 0.47 |
|  | Disability -> No disability | 1.0 | (0.6, | 1.6) | 0.89 | 0.9 | (0.6, | 1.3) | 0.59 |
|  | Disability -> Death | 0.8 | (0.7, | 1.0) | 0.01 | 0.7 | (0.6, | 0.8) | <0.001 |
| Cognitive | No disability -> Disability | 1.3 | (1.0, | 1.7) | 0.05 | 1.8 | (1.3, | 2.5) | <0.001 |
| impairment | No disability -> Death | 1.4 | (0.9, | 2.2) | 0.14 | 0.9 | (0.4, | 2.2) | 0.81 |
|  | Disability -> No disability | 0.5 | (0.3, | 0.8) | 0.006 | 0.5 | (0.3, | 0.7) | 0.001 |
|  | Disability -> Death | 1.1 | (0.9, | 1.3) | 0.31 | 1.2 | (1.0, | 1.4) | 0.03 |
| Coronary | No disability -> Disability | 1.8 | (1.4, | 2.4) | <0.001 | 1.4 | (1.1, | 1.9) | 0.02 |
| Heart | No disability -> Death | 1.7 | (1.1, | 2.6) | 0.02 | 1.2 | (0.7, | 2.1) | 0.52 |
| Disease | Disability -> No disability | 1.9 | (1.1, | 3.1) | 0.02 | 1.0 | (0.7, | 1.5) | 0.94 |
|  | Disability -> Death | 1.1 | (0.9, | 1.3) | 0.31 | 1.0 | (0.8, | 1.2) | 0.79 |
| Diabetes | No disability -> Disability | 1.4 | (0.8, | 2.3) | 0.21 | 1.4 | (1.0, | 1.9) | 0.04 |
|  | No disability -> Death | 1.7 | (0.8, | 3.3) | 0.14 | 1.6 | (0.9, | 2.8) | 0.10 |
|  | Disability -> No disability | 0.9 | (0.4, | 2.2) | 0.81 | 1.0 | (0.6, | 1.5) | 0.97 |
|  | Disability -> Death | 1.1 | (0.8, | 1.6) | 0.59 | 1.2 | (1.0, | 1.5) | 0.08 |
| Hearing | No disability -> Disability | 1.4 | (1.0, | 1.8) | 0.02 | 1.4 | (1.1, | 1.9) | 0.02 |
| difficulties | No disability -> Death | 0.8 | (0.5, | 1.4) | 0.40 | 0.7 | (0.3, | 1.3) | 0.34 |
|  | Disability -> No disability | 1.2 | (0.7, | 2.2) | 0.53 | 1.1 | (0.8, | 1.7) | 0.62 |
|  | Disability -> Death | 1.1 | (0.9, | 1.3) | 0.31 | 1.2 | (1.0, | 1.4) | 0.03 |
| Peripheral | No disability -> Disability | 1.5 | (1.0, | 2.5) | 0.08 | 1.3 | (0.8, | 1.9) | 0.23 |
| Vascular | No disability -> Death | 1.6 | (0.8, | 3.2) | 0.18 | 2.1 | (1.2, | 3.8) | 0.01 |
| Disease | Disability -> No disability | 1.1 | (0.5, | 2.5) | 0.82 | 1.1 | (0.7, | 1.7) | 0.67 |
|  | Disability -> Death | 0.9 | (0.7, | 1.3) | 0.50 | 0.9 | (0.7, | 1.1) | 0.36 |
| Respiratory | No disability -> Disability | 1.4 | (1.1, | 1.9) | 0.02 | 1.4 | (1.0, | 2.0) | 0.06 |
| difficulties | No disability -> Death | 1.6 | (1.1, | 2.5) | 0.02 | 0.6 | (0.2, | 1.7) | 0.35 |
|  | Disability -> No disability | 0.7 | (0.4, | 1.3) | 0.24 | 1.2 | (0.8, | 1.8) | 0.38 |
|  | Disability -> Death | 1.3 | (1.0, | 1.5) | 0.01 | 1.3 | (1.1, | 1.6) | 0.006 |
| Stroke | No disability -> Disability | 3.1 | (2.1, | 4.6) | <0.001 | 2.0 | (1.3, | 3.0) | 0.001 |
|  | No disability -> Death | 1.1 | (0.3, | 4.1) | 0.89 | 0.8 | (0.2, | 3.3) | 0.76 |
|  | Disability -> No disability | 0.5 | (0.3, | 1.1) | 0.04 | 0.6 | (0.3, | 0.9) | 0.07 |
|  | Disability -> Death | 1.3 | (1.0, | 1.6) | 0.03 | 1.1 | (0.9, | 1.3) | 0.31 |
| Vision | No disability -> Disability | 1.5 | (1.0, | 2.2) | 0.04 | 1.4 | (1.0, | 1.9) | 0.04 |
| impairment | No disability -> Death | 1.1 | (0.5, | 2.3) | 0.81 | 0.5 | (0.2, | 1.3) | 0.15 |
|  | Disability -> No disability | 0.7 | (0.3, | 1.5) | 0.38 | 0.6 | (0.3, | 1.0) | 0.10 |
|  | Disability -> Death | 1.0 | (0.8, | 1.3) | 0.79 | 1.1 | (0.9, | 1.3) | 0.31 |

**Table H:** Relative Risk Ratios (RRR) for transition with each long-term condition (relative to without condition) from unadjusted models for women in the Cognitive Function and Ageing Studies (CFAS I and CFAS II), with 95% confidence intervals (95% CI) and p-values (p).

|  |  | **CFAS I** | | | | **CFAS II** | | | |
| --- | --- | --- | --- | --- | --- | --- | --- | --- | --- |
|  |  | **RRR** | **95% CI** | | **p** | **RRR** | **95% CI** | | **p** |
| Arthritis | No disability -> Disability | 1.8 | (1.5, | 2.2) | <0.001 | 1.6 | (1.3, | 2.0) | <0.001 |
|  | No disability -> Death | 0.7 | (0.4, | 1.3) | 0.24 | 0.6 | (0.2, | 1.3) | 0.28 |
|  | Disability -> No disability | 1.4 | (1.0, | 2.2) | 0.09 | 0.9 | (0.7, | 1.3) | 0.50 |
|  | Disability -> Death | 0.7 | (0.6, | 0.8) | <0.001 | 0.9 | (0.8, | 1.0) | 0.06 |
| Cognitive | No disability -> Disability | 1.2 | (1.0, | 1.4) | 0.03 | 1.5 | (1.2, | 1.9) | <0.001 |
| impairment | No disability -> Death | 1.1 | (0.5, | 2.3) | 0.81 | 1.0 | (0.3, | 3.1) | 0.95 |
|  | Disability -> No disability | 0.7 | (0.5, | 1.0) | 0.04 | 0.6 | (0.4, | 0.8) | 0.004 |
|  | Disability -> Death | 1.3 | (1.2, | 1.5) | <0.001 | 1.2 | (1.1, | 1.4) | 0.003 |
| Coronary | No disability -> Disability | 2.0 | (1.6, | 2.6) | <0.001 | 1.5 | (1.1, | 2.0) | 0.008 |
| Heart | No disability -> Death | 2.8 | (1.3, | 5.8) | 0.007 | 3.0 | (1.3, | 6.7) | 0.009 |
| Disease | Disability -> No disability | 1.2 | (0.8, | 1.7) | 0.34 | 1.1 | (0.8, | 1.6) | 0.59 |
|  | Disability -> Death | 0.8 | (0.7, | 0.9) | <0.001 | 0.9 | (0.8, | 1.0) | 0.06 |
| Diabetes | No disability -> Disability | 1.6 | (1.1, | 2.3) | 0.01 | 1.6 | (1.2, | 2.2) | 0.002 |
|  | No disability -> Death | 2.8 | (1.1, | 7.2) | 0.03 | 1.6 | (0.5, | 5.2) | 0.43 |
|  | Disability -> No disability | 0.5 | (0.2, | 1.0) | 0.09 | 0.8 | (0.5, | 1.2) | 0.32 |
|  | Disability -> Death | 0.9 | (0.7, | 1.2) | 0.44 | 1.1 | (0.9, | 1.3) | 0.31 |
| Hearing | No disability -> Disability | 1.1 | (0.9, | 1.3) | 0.31 | 1.0 | (0.8, | 1.4) | 0.75 |
| difficulties | No disability -> Death | 0.9 | (0.4, | 2.3) | 0.81 | 1.1 | (0.4, | 3.0) | 0.85 |
|  | Disability -> No disability | 0.9 | (0.6, | 1.5) | 0.65 | 0.8 | (0.6, | 1.2) | 0.21 |
|  | Disability -> Death | 0.9 | (0.8, | 1.1) | 0.19 | 1.1 | (0.9, | 1.2) | 0.19 |
| Peripheral | No disability -> Disability | 1.9 | (1.1, | 3.1) | 0.02 | 1.3 | (0.9, | 1.9) | 0.17 |
| Vascular | No disability -> Death | 2.2 | (0.6, | 8.6) | 0.25 | 1.5 | (0.4, | 6.1) | 0.56 |
| Disease | Disability -> No disability | 1.5 | (0.8, | 2.8) | 0.20 | 0.7 | (0.5, | 1.1) | 0.08 |
|  | Disability -> Death | 0.7 | (0.5, | 0.9) | 0.02 | 0.7 | (0.6, | 0.9) | <0.001 |
| Respiratory | No disability -> Disability | 1.5 | (1.2, | 1.9) | <0.001 | 1.3 | (1.0, | 1.7) | 0.05 |
| difficulties | No disability -> Death | 1.1 | (0.5, | 2.5) | 0.82 | 1.6 | (0.7, | 3.8) | 0.28 |
|  | Disability -> No disability | 0.9 | (0.6, | 1.3) | 0.59 | 0.7 | (0.5, | 1.0) | 0.04 |
|  | Disability -> Death | 0.9 | (0.8, | 1.1) | 0.19 | 1.0 | (0.9, | 1.2) | 0.54 |
| Stroke | No disability -> Disability | 1.6 | (1.1, | 2.4) | 0.02 | 1.7 | (1.0, | 2.7) | 0.04 |
|  | No disability -> Death | 2.4 | (0.7, | 8.4) | 0.17 | 2.1 | (0.2, | 18.1) | 0.52 |
|  | Disability -> No disability | 0.5 | (0.3, | 0.9) | 0.01 | 1.1 | (0.7, | 1.8) | 0.69 |
|  | Disability -> Death | 1.4 | (1.2, | 1.7) | <0.001 | 1.2 | (1.0, | 1.4) | 0.03 |
| Vision | No disability -> Disability | 1.5 | (1.2, | 2.0) | 0.002 | 1.3 | (1.0, | 1.8) | 0.08 |
| impairment | No disability -> Death | 2.2 | (1.0, | 5.2) | 0.06 | 1.4 | (0.5, | 4.0) | 0.53 |
|  | Disability -> No disability | 1.2 | (0.8, | 2.0) | 0.44 | 0.8 | (0.5, | 1.3) | 0.36 |
|  | Disability -> Death | 0.9 | (0.8, | 1.0) | 0.06 | 1.1 | (0.9, | 1.2) | 0.19 |
